# Differential Expression Levels of Sox9 in Early Neocortical Radial Glial Cells Regulate the Decision between Stem Cell Maintenance and Differentiation

**DOI:** 10.1523/JNEUROSCI.2905-20.2021

**Published:** 2021-08-18

**Authors:** Jaime Fabra-Beser, Jessica Alves Medeiros de Araujo, Diego Marques-Coelho, Loyal A. Goff, Marcos R. Costa, Ulrich Müller, Cristina Gil-Sanz

**Affiliations:** ^1^BIOTECMED Institute, Universidad de Valencia, Burjassot, 46100 Valencia, Spain; ^2^The Solomon H. Snyder Department of Neuroscience, Johns Hopkins University School of Medicine, Baltimore, Maryland 21205; ^3^Brain Institute, Federal University of Rio Grande do Norte, RN 59056-450, Natal, Brazil; ^4^Bioinformatics Multidisciplinary Environment, IMD, Federal University of Rio Grande do Norte, RN 59078-400, Natal, Brazil; ^5^Institut National de la Santé et de la Recherche Médicale U1167-RID-AGE facteurs de risque et déterminants moléculaires des maladies liés au vieillissement, DISTALZ, Centre Hospitalier Universitaire de Lille, Institut Pasteur de Lille, 59000 Lille, France

**Keywords:** cortical development, progenitors diversity, radial glia cells, Sox9

## Abstract

Radial glial progenitor cells (RGCs) in the dorsal telencephalon directly or indirectly produce excitatory projection neurons and macroglia of the neocortex. Recent evidence shows that the pool of RGCs is more heterogeneous than originally thought and that progenitor subpopulations can generate particular neuronal cell types. Using single-cell RNA sequencing, we have studied gene expression patterns of RGCs with different neurogenic behavior at early stages of cortical development. At this early age, some RGCs rapidly produce postmitotic neurons, whereas others self-renew and undergo neurogenic divisions at a later age. We have identified candidate genes that are differentially expressed among these early RGC subpopulations, including the transcription factor Sox9. Using *in utero* electroporation in embryonic mice of either sex, we demonstrate that elevated Sox9 expression in progenitors affects RGC cell cycle duration and leads to the generation of upper layer cortical neurons. Our data thus reveal molecular differences between progenitor cells with different neurogenic behavior at early stages of corticogenesis and indicates that Sox9 is critical for the maintenance of RGCs to regulate the generation of upper layer neurons.

**SIGNIFICANCE STATEMENT** The existence of heterogeneity in the pool of RGCs and its relationship with the generation of cellular diversity in the cerebral cortex has been an interesting topic of debate for many years. Here we describe the existence of RGCs with reduced neurogenic behavior at early embryonic ages presenting a particular molecular signature. This molecular signature consists of differential expression of some genes including the transcription factor Sox9, which has been found to be a specific regulator of this subpopulation of progenitor cells. Functional experiments perturbing expression levels of Sox9 reveal its instructive role in the regulation of the neurogenic behavior of RGCs and its relationship with the generation of upper layer projection neurons at later ages.

## Introduction

The neocortex is evolutionarily the most recent addition to the brain and executes complex tasks including sensory integration and cognition. Neocortical development depends on precisely orchestrated mechanisms ranging from the generation of the neural cells in specialized germinal zones, to their migration to defined locations and the establishment of specific connections. During cortical development radial glial progenitor cells (RGCs) generate different subtypes of excitatory neurons that will ultimately form the layers of the cerebral cortex and make connections to distinct subcortical and cortical targets. The mature cortex consists of six principal layers that are generated following a temporal sequence. Glutamatergic neurons that populate deep layers of the cortex are generated first, followed by upper layer (UL) neurons and finally glia cells (Molyneaux et al., 2007).

Multiple progenitor subtypes with distinct morphologies and behaviors have been described in the neocortex. RGCs occupy the ventricular zone (VZ) and are connected to the ventricular and pial surfaces of the neocortex by apical and basal processes, respectively. Intermediate progenitor (IP) cells are basal progenitors grouped in the subventricular zone (SVZ) and are mostly generated by asymmetric divisions of RGCs (Miyata et al., 2004; Noctor et al., 2004). Other types of progenitor cells have been described more recently, including short neural precursors (Gal et al., 2006) and outer RGCs (oRGCs; Fietz et al., 2010; Hansen et al., 2010; Reillo et al., 2011). oRGCs are expanded in gyrencephalic animals and are thought to be important for the expansion of neocortical neurons in primates, especially those occupying the enlarged upper cortical layers (Lui et al., 2011; LaMonica et al., 2012).

Despite the advance of knowledge in recent years concerning the identification of morphologic differences among neocortical progenitors, the mechanisms that regulate neuronal progenitor diversification are not well understood. In fact, the extent of neocortical progenitor heterogeneity is still unclear as well as how these progenitor cells are able to generate the diversity of neocortical cells. The traditional hypothesis states that the fate of principal neurons is determined by its birth date, suggesting the existence of a common RGC progenitor that becomes progressively restricted with developmental time (McConnell and Kaznowski, 1991; Frantz and McConnell, 1996; Desai and McConnell, 2000; Shen et al., 2006; Gao et al., 2014). However, recent studies suggest that the pool of RGCs is more diverse, with some RGCs primarily generating certain types of cells including upper layer cortical glutamatergic neurons and B1 cells (Franco et al., 2012; Fuentealba et al., 2015; García-Moreno and Molnár, 2015; Gil-Sanz et al., 2015; Llorca et al., 2019). Nevertheless, the mechanisms that might lead to progenitor diversification are unclear, and molecular markers that would distinguish disparate populations of RGCs are largely unknown.

To identify molecular differences between RGC progenitors, we used an *in utero* electroporation (IUE) approach to introduce plasmid constructs containing different promoters driving the expression of reporter genes, based on the prediction that different promoters may be active in distinct neocortical RGC subtypes, as described previously (García-Moreno and Molnár, 2015). Using this strategy, we identified RGCs with reduced neurogenic behavior at early embryonic ages that eventually produce predominantly corticocortical projection neurons occupying upper neocortical cell layers. Using single cell RNA sequencing (scRNA seq), we characterized the molecular signature of these RGC progenitors, revealing significantly higher levels of Sox9 expression compared to other RGCs. Moreover, using transcriptional regulatory network analysis we identified the role of Sox9 as a specific regulator of these RGCs. Notably, Sox9 is a transcription factor (TF) involved in stem cell maintenance (Scott et al., 2010; Liu et al., 2016) and associated with progenitor quiescence inside and outside the nervous system (Kadaja et al., 2014; Llorens-Bobadilla et al., 2015). Using functional perturbations, we demonstrate that Sox9 expression levels are critical to control the cell cycle duration of a subset of RGCs, which remain in the VZ during extended periods of time, to produce upper layer neocortical neurons at later developmental stages.

## Materials and Methods

### 

#### Mice

All experiments involving mice were conducted in accordance with Spanish legislation as well as with European Union Directive 2010/63/EU and US animal welfare regulations; and were approved by the ethical committee of the University of Valencia and the Conselleria de Agricultura, Desarrollo Rural, Emergencia Climática y Transición Ecológica of the Comunidad Valenciana, and US Institutional Animal Care and Use Committees.

C57BL/6 wild-type and Ai9 Cre reporter strains (Madisen et al., 2010) used for experimentation were originally obtained from The Jackson laboratory and are currently being bred in the animal facilities of the University of Valencia, Burjassot. Both female and male mice were analyzed in this study. The induction of recombination in *Ai9* mice electroporated (EP) with Cre-inducible plasmids was achieved by intraperitoneal injection of pregnant dams with the faster-acting and shorter-lived tamoxifen metabolite 4-hydroxy-tamoxifentamoxifen [4-OHT; Sigma-Aldrich; 1 mg/20 g body weight, dissolved as described previously (Guenthner et al., 2013)].

#### Expression constructs

Hes5-dsRed (plasmid #26868, Addgene) was generated by Nicholas Gaiano, Johns Hopkins University School of Medicine (Mizutani et al., 2007). Hes5-GFP was generated by replacing dsRed2 with EGFP in Hes5-DsRed (Franco et al., 2012). CAG-blue fluorescent protein (BFP; plasmid #127348, Addgene) was deposited by Phil Sharp, Massachusetts Institute of Technology (Guo et al., 2019). CAG-monomeric RFP (mRFP; plasmid #32600, Addgene) was deposited by Anna-Katerina Hadjantonakis, Memorial Sloan Kettering Cancer Center (Long et al., 2005. CAG-mRFP;Hes5p-GFP was generated by subcloning CAGmRFP and a polyadenylation signal into Hes5-GFP. CAG-CreERT2 (plasmid #14797, Addgene) was deposited by Connie Cepko, Harvard Medical School (Matsuda and Cepko, 2007). Hes5-CreERT2 was generated by replacing CAG with the Hes5 promoter. PCIG (plasmid #11159, Addgene) was deposited by Connie Cepko, Harvard Medical School (Matsuda and Cepko, 2004). CBFRE-GFP (plasmid #17705, Addgene) was deposited by Nicholas Gaiano, Johns Hopkins University School of Medicine (Mizutani et al., 2007). TOP-GFP (plasmid #35489, Addgene) was deposited by Ramesh Shivdasani, Dana-Farber Cancer Institute (Horst et al., 2012). Emx2-GFP was generated according to the study by García-Moreno and Molnár (2015). PCIG-FL Sox9 was generated by cloning the Sox9 coding sequence into PCIG. sc-shRNA (pLKO.1-shSCR; plasmid #17920, Addgene) was deposited by Sheila Stewart, Washington University School of Medicine (Saharia et al., 2008). S9-shRNA (pLKO.1-sh-mSOX9-2; plasmid #40645, Addgene) was deposited by Bob Weinberg, Whitehead Institute for Biomedical Research (Guo et al., 2012).

#### *In utero* electroporation

*In utero* electroporation was performed as described previously (Gil-Sanz et al., 2013). Briefly, timed pregnant mice were anesthetized with isoflurane. Once the pedal reflex was lost, analgesic solution was injected subcutaneously. The abdominal area was shaved, and an incision in the abdomen of the mouse was performed to expose the uterine horns. Endotoxin-free plasmid DNA solution (0.5–1 µl) was injected into one of the lateral ventricles of the embryo. For electroporation of embryonic day 12.5 (E12.5) embryos, five pulses of 30 V were delivered, spaced by 950 ms. After electroporation, the uterus was placed in the abdominal cavity, and the abdominal wall and skin were sutured. Embryos were left to develop *in utero* for the indicated time or females were allowed to give birth and pups were killed at the indicated age.

#### *In utero* FlashTag injection

Carboxyfluorescein succinimidyl ester or FlashTag (FT) injections were performed at E14.5, as described previously (Govindan et al., 2018), on previously electroporated embryos at E12.5. As described for *in utero* electroporation, E14.5 pregnant females were anesthetized with isoflurane, analgesic solution was injected, and abdominal incision was performed to expose the uterine horns. 0.5 µl of 10 mm FT (CellTrace CFSE Cell Proliferation Kit, catalog #C34554, Thermo Fisher Scientific) was injected into one of the lateral ventricles of the embryo. The uterus was then placed in the abdominal cavity, the abdominal wall was sutured, and embryos were allowed to develop *in utero* until collection time at E18.5.

#### Immunohistochemistry

##### Tissue processing.

Embryonic brains were dissected in a PBS solution, fixed in 4% paraformaldehyde (PFA) in PBS overnight at 4°C, and later stored in PBS-0.05% sodium azide at 4°C. After embedding in 4% low-melting point agarose in PBS, brains were sectioned coronally at 100 µm using a vibratome (catalog #VT1200S, Leica). Postnatal mice were transcardially perfused with 4% PFA before dissection and postfixation. Vibratome coronal sections were performed obtaining 60 µm sections.

##### Immunofluorescence on brain sections.

Brain sections were incubated for 1 h in blocking solution (10% horse serum, 0.2% Triton X-100 in PBS) at room temperature. Sections were incubated overnight with the primary antibody at the appropriated dilution in blocking solution at 4°C. Sections were washed three times with PBS and incubated for 1 h with the respective secondary antibodies (1:1000) in blocking solution at room temperature. After three washes in PBS, sections were mounted on slides with Fluoromount G (Electron Microscopy Sciences).

##### Antibodies.

The following antibodies were used: rabbit anti-bromodeoxyuridine (1:1000; catalog #12304, Megabase Research Products); rat anti-Ctip2 (1:500; catalog #ab18465, Abcam); rabbit anti-Cux1 (1:300; catalog #sc-13 024, Santa Cruz Biotechnology); rabbit anti-Cux1 (1:1000; catalog #11733–1-Apr, Proteintech); rabbit anti-doublecortin (DCX; 1:1000; catalog #ab18723, Abcam); goat anti-GFP (1:500; catalog #600–101-215 M, Rockland); rabbit anti-Ki67 (1:500; catalog #AB9260, EMD); goat anti-Mcm2 (minichromosomal maintenance protein 2; 1:250; catalog #sc-9839, Santa Cruz Biotechnology); rabbit anti-Pax6 (1:500; catalog #GTX113241, GeneTex); mouse anti-phospho vimentin (1:500; catalog #D076-3, MBL); mouse anti-RFP (1:200; catalog #LS-C29691, LSBio); rabbit anti-Satb2 (1:2000; catalog #ab92446, Abcam); rabbit anti-Sox9 (1:1000; catalog #AB184547, Abcam); rabbit anti-Tle4 (1:500; catalog #ab140485, Abcam); and rabbit anti-Tbr2 (Eomes; 1:500; catalog #AB23345, Abcam). Fluorescent secondary antibodies were used at 1:1000 (Jackson ImmunoResearch).

##### Imaging.

Images were acquired using a C2 (Nikon) or a FluoView FV10i (Olympus) confocal laser-scanning microscope. Image analysis and quantification were performed using Photoshop (Adobe), FIJI (ImageJ), and R software.

#### Quantification of tissue sections

Four different animals were used for most of the conditions, with a couple of exceptions where three animals were analyzed. Several rostrocaudal sections were selected based on the electroporation location, limiting the use of electroporations to the lateral neocortex including the somatosensory neocortex.

##### Analysis of the developing neocortex at E13.5.

Quantifications in the developing neocortex were performed by dividing its surface in two regions: the VZ and outside the VZ [including the SVZ, intermediate zone (IZ), and cortical plate (CP)]. Density and morphologic features were used to differentiate these regions.

##### Analysis of the ventricular zone at E14.0.

Quantifications were performed by delimiting the VZ using cellular density and morphologic features. Additionally, for cell distribution analysis, the VZ was divided in two equally sized regions: the inner VZ (IVZ), half in contact with the surface of the ventricle, and the outer VZ (OVZ), half in contact with the SVZ.

##### Analysis of the developing neocortex at E14.5.

Quantifications in the developing neocortex were performed by dividing its surface in four regions: VZ, SVZ, IZ, and CP. Cellular density and morphologic features were used to differentiate these regions. Furthermore, quantifications using cell type markers Pax6, Tbr2, and Dcx were conducted.

##### Analysis of the cortical plate at E18.5 and postnatal day 0.

Quantifications were performed by dividing the cortical plate in 10 equal-sized regions, which are referred to as bins. The bin division was used since the cortical plate was still unmatured and some neurons are still migrating at this stage.

##### Analysis of the cortical plate at postnatal days 12 and 20.

Quantifications of the cortical plate were performed by dividing the neocortex surface into two regions: UL and lower layer (LL). To differentiate these two regions, immunochemistry with Cux1 antibody was used to define the UL (II–IV).

#### Tissue preparation for single-cell sorting and sequencing

Brains electroporated with the CAG-mRFP;Hes5-GFP plasmid were dissected 11 h after electroporation and embedded in 4% agarose in complete HBSS medium (Polleux and Ghosh, 2002) and sectioned using a vibratome (model VT1200S, Leica). Electroporated areas of the lateral cortex were microdissected under the fluorescent dissecting scope (Axio Zoom, Zeiss). Tissue was dissociated into single cells using the Papain Dissociation System (catalog #LK0031500, Worthington). Resuspended cells were processed using a cell sorter (model XDP, MoFlo), and viable single cells expressing mRFP (CAG^+^) or mRFP and GFP (Hes5^+^) were individually collected in 96-well plates with lysis buffer. Individual lysed collected cells were processed for scRNA-seq using a modified Smart-seq2 protocol (Chevée et al., 2018). Data were aligned to mouse reference genome (mm10) using Hisat2 (Kim et al., 2015) and were quantified against a modified reference transcriptome (GENCODE version M15; Frankish et al., 2019) with cuffquant (Trapnell et al., 2014). Default parameters were set for both programs except for parallelization value (–p 6) to improve processing time.

#### scRNA-seq analysis

Data were analyzed using Seurat R package version 2 (Butler et al., 2018). To import data into a Seurat object and eliminate low-quality cells/genes (quality check), CreateSeuratObject function selected cells with at least 300 genes, and genes that were expressed in at least three cells. A global scaling normalization method (LogNormalize by 10^4^ factor) was implemented using NormalizeData. Highly variable genes were detected (FindVariableGenes) across the single cells and data were scaled (ScaleData) to linear transform our data and regress out variables that would not give meaningful information to our analysis such as a high percentage of mitochondrial genes expression and number of molecules detected. RunPCA was used to perform linear dimensional reduction, and among those principal component analyses (PCAs), 10 (based on PCElbowPlot) were selected for further analysis. Clusters and t-distributed stochastic neighbor embedding (tSNE) two-dimensional visualizations were built using PCAs with FindClusters and RunTSNE functions, respectively. FindAllMarkers was used to identify differentially expressed genes (DEGs) in each group, and clusters were renamed based on the expression of identified known cell markers. Statistically significant DEGs were identified using the nonparametric Wilcoxon rank-sum test (significance thresholds: log2-fold change > 0.3; adjusted *p* value < 0.05).

#### Transcriptional regulatory networks

We used the Reconstruction of Transcriptional Networks package (Castro et al., 2016) to generate a gene regulatory network (GRN) based on scRNA-seq data, as previously described (Jackson et al., 2020). Specifically, we used scRNA-seq data generated from the mouse dorsal telencephalon at E13 (Yuzwa et al., 2017). This GRN defines regulons (possible target genes) for a set of curated transcription factors. The regulon for each TF is composed of all the genes whose expression data display significant mutual information with those of a given transcription factor and are therefore likely to be regulated by that transcription factor (Castro et al., 2016; Jackson et al., 2020). To select mouse TFs, a transcription factor database (TcoF-DB, version 2; Schmeier et al., 2017) was used to identify TFs in our data. Then, TNI-class object was created using previously described inputs (normalized table and selected TFs: tni.constructor), followed by permutation analysis (tni.permutation: nPermutations = 1000) and bootstrap (tni.bootstrap). To assess whether a gene list is enriched in a given regulon, we used the master regulator analysis (Carro et al., 2010; Castro et al., 2016). A significant enrichment (adjusted *p* value < 0.01, Fisher's exact test) indicates that the TF controlling the regulon is likely to be involved in the regulation of the gene list and, therefore, can be considered as master regulator. Graphical representations of the regulon network were generated using RedeR package (Castro et al., 2016).

#### Mean fluorescence intensity of Sox9

Immunostaining with Sox9 antibody (1:1000; catalog #AB184547, Abcam) was performed on slices from E12.5 electroporated embryos. For CAG-mRFP;Hes5-GFP electroporation analysis, brains were collected 11 h postelectroporation (E13.0), whereas for short-timed knock-down (KD) experiments brains were analyzed 48 h postelectroporation (E14.5). Images were obtained using a FluoView FV10i (Olympus) confocal laser-scanning microscope and were analyzed using FIJI software. The mean fluorescence intensity (MFI) was measured for electroporated cells in the VZ with RGC morphology by selecting each nucleus using the polygon selection tool. Cells from at least two sections from each animal were analyzed. MFI was measured on a scale of 0–255 arbitrary units (a.u.). The MFI for every counted cell was corrected by its particular background value, measured for every counted section, as follows: [(${\text{cell intensity}}_{n_{x}}$–${\text{background}}_{x}$)/(255 – ${\text{background}}_{x}$)] * 100.

Bar graph represents the mean of MFI calculated for each animal. The distribution of Sox9 intensity of fluorescence graph represents the frequency in percentage of cells regarding the intensity of fluorescence in arbitrary units.

#### Cell cycle length analysis

##### The 30 min EdU pulse.

*In utero* electroporation was performed at E12.5 using PCIG and PCIG-FL Sox9 constructs. At 36 h postelectroporation (E14.0), pregnant dams were injected with 50 mg/kg 5-etinil-2'-desoxiuridina (EdU). Embryos were collected at 30 min post-EdU injection. Brain sections were stained for EdU (Click-it Plus EdU, Thermo Fisher Scientific).

##### The 24 h BrdU pulse.

*In utero* electroporation was performed at E12.5 using PCIG and PCIG-FL Sox9 constructs. At 12 h postelectroporation, pregnant dams were injected with 50 mg/kg BrdU. Embryos were collected 24 h post-BrdU injection. Brain sections were stained for BrdU.

##### Dual-pulse labeling.

The mean of the durations of total cell cycle (Tc) and total S phase (Ts) was calculated for E14.0 EP cells in the VZ using a dual-pulse labeling strategy modified from the study by Harris et al. (2018). In brief, *in utero* electroporation was performed at E12.5 using PCIG and PCIG-FL Sox9 constructs. At 36 h postelectroporation, pregnant dams were injected with 50 mg/kg BrdU, and 1 h later with 50 mg/kg EdU. One-half hour after EdU injection, embryos were collected and immersion fixed in 4% PFA. Brain sections were stained for BrdU and EdU. For quantification, we identified GFP^+^ cells located in the VZ with RGC morphology. The Ts was calculated as the fraction of cells that remain in S phase (BrdU^+^EdU^+^) to the cells that leave S phase before EdU injection (BrdU^+^EdU^–^), multiplied by the injection interval (1 h). The Tc was calculated as the Ts divided by the fraction of cells in the VZ that are in S phase [EdU^+^/(GFP^+^)], corrected by the proportion of EP cell in the VZ that are in cell cycle, referred to as the growth fraction (GF), as follows:

$$\text{Ts}=\frac{{\text{BrdU}}^{\text{+}}{\text{EdU}}^{+}}{{\text{BrdU}}^{\text{+}}{\text{EdU}}^{\text{-}}};\text{ Tc}=\frac{Ts}{\left({\text{EdU}}^{\text{+}}{\text{/(GFP}}^{\text{+}}\text{ × GF})\right)};\text{ GF}=\frac{{\text{GFP}}^{\text{+}}{\text{Antibody}}^{\text{+}}}{{\text{GFP}}^{\text{+}}}.$$

#### Experimental design and statistical analysis

Mean, SD, SEM, and statistical analysis were performed using Excel from Microsoft. Statistically significant differences of means were assessed by Student's unpaired *t* test comparing two groups. A *p* value < 0.05 was considered a significant difference (*). The exact *p* value, mean ± SEM, and *n* are reported in the Results section.

## Results

### *In utero* electroporation using different promoters as a strategy to distinguish progenitors with different neurogenic behavior

**Figure 1.**
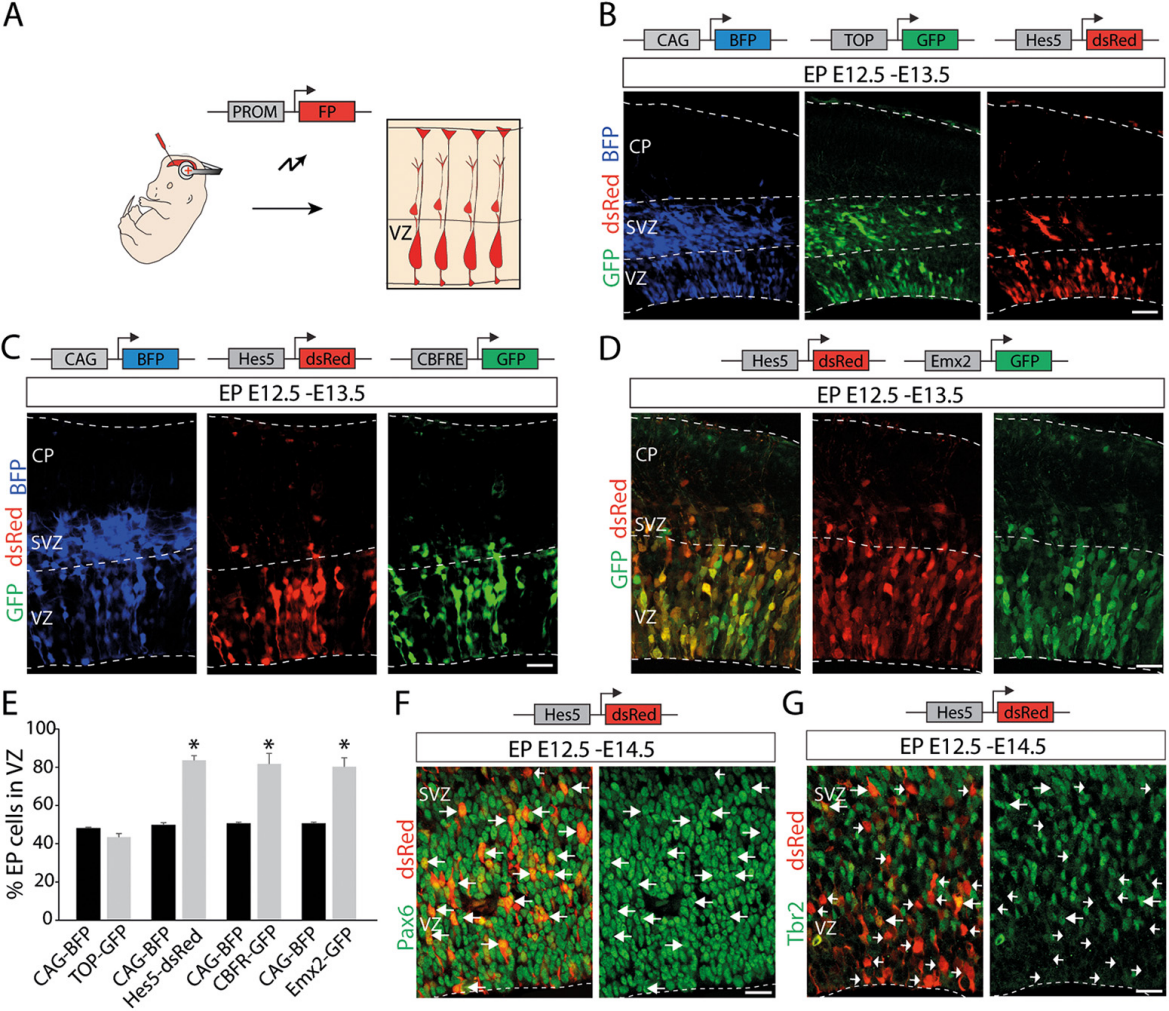
*In utero* electroporation strategy to identify RGC subpopulations. ***A***, Schematics of the electroporation strategy for labeling different subpopulations of RGCs. PROM, Promoter; FP, fluorescence protein. ***B***, Triple electroporation at E12.5 of CAG-BFP (blue), Wnt reporter plasmid TOP-GFP (green), and Notch reporter plasmid Hes5-dsRed (red), with analysis at E13.5. Note the similar distribution of labeled cells using the general promoter (CAG), and the Wnt reporter plasmid showing a notorious number of cells in the SVZ. The Notch reporter plasmid allowed the labeling of cells mostly present in the VZ. ***C***, Triple electroporation at E12.5 of CAG-BFP (blue), Notch reporter plasmids Hes5-dsRed (red), and CBFRE-GFP (green), with analysis at E13.5. Note that CBFF-RE and Hes5 promoter fragments drive a similar labeling pattern. ***D***, Coelectroporation of Hes5-dsRed (red) and Emx2-GFP (green) at E12.5, with analysis at 13.5. Note the virtual colocalization of both reporter proteins in cells activating these promoter fragments. ***E***, Quantification (mean ± SEM) of EP cells in the VZ at 13.5 with different expression vectors exhibiting differences in their neurogenic behavior. *p < 0.002. ***F***, ***G***, Immunostaining (green) at E14.5 for Pax6 (***F***) and Tbr2 (***G***) of E12.5 Hes5-dsRed (red) electroporated embryos. ***F***, Arrows point examples of dsRed^+^/Pax6^+^ cells. ***G***, Examples of dsRed^+^/Tbr2^+^ cells (arrows) and dsRed^+^/Tbr2^–^ cells (arrowheads). FP, Fluorescent protein; PROM, promoter fragment. Scale bars, 50 µm.

It has been reported that RGCs targeted with constructs using the *Emx2* enhancer/promoter at E12.5 showed delayed neurogenic divisions compared with RGCs targeted with ubiquitous promoters (García-Moreno and Molnár, 2015). We used this strategy to identify progenitors with different neurogenic behavior, and we conducted the experiments on the same embryonic day (E12.5) reported in the earlier study. We selected expression vectors containing promoter fragments driving fluorescent proteins that report the activity of important molecular pathways involved in the development of the CNS including Wnt signaling (Hirabayashi et al., 2004; Chenn, 2008) and Notch signaling (Yoon and Gaiano, 2005; Louvi and Artavanis-Tsakonas, 2006). We compared the labeling observed using these promoters to that of the general promoter CAG driving BFP (Fig. 1*A*). To report Wnt signaling, we used the TOP-GFP plasmid containing a tandem of seven TCF/LEF promoter elements (Horst et al., 2012). To report Notch activity, we selected a plasmid containing the Hes5 promoter fragment driving dsRed protein expression (Mizutani et al., 2007). We performed *in utero* electroporation using the three plasmids to visualize possible differences in the types of cells activating the different promoters in the same tissue. Twenty-four hours after electroporation using the CAG promoter, we found a considerable number of labeled cells located in the SVZ-IZ. A similar result was observed using the Wnt reporter plasmid, showing no significant differences in the percentage of electroporated cells inside the VZ (CAG-BFP, 47.97 ± 1.23%; TOP-GFP, 43.41 ± 2.45%; *t*_(6)_ = −1.67, *p* = 0.147, unpaired *t* test; *n* = 4; Fig. 1*B*,*E*). However, with the Hes5 reporter most of the cells were found in the VZ (CAG-BFP, 50.51 ± 0.66%; Hes5-dsRed, 81.69 ± 5.47%; *t*_(6)_ = 5.66, *p* = 0.001, unpaired *t* test; *n* = 4; Fig. 1*B*,*E*). A similar pattern was observed using another Notch reporter plasmid containing the promoter CBF-RE (Mizutani et al., 2007), a tandem of five CBF1 binding elements driving the expression of a different fluorescent protein, GFP (pCAG-BFP, 49.67 ± 0.69%; CBFRE-GFP, 83.55 ± 1.91%; *t*_(6)_ = 16.71, *p* = 2.94 × 10^−6^, unpaired *t* test; *n* = 4; Fig. 1*C*,*E*). Coelectroporation experiments of Hes5-dsRed and Emx2-GFP plasmids at E12.5 showed colocalization of the two reporter genes in all of the targeted cells 24 h after electroporation (Fig. 1*D*,*E*). Comparison of the distribution of labeled cells using the Emx2 and CAG promoters consistently revealed the existence of statistically significant differences (CAG-BFP, 50.51 ± 0.66%; Emx2-GFP, 80.13 ± 4.75%; *t*_(6)_ = 6.17, *p* = 0.00,083, unpaired *t* test; *n* = 4; Fig. 1*D*,*E*). For simplicity, we will refer to cells activating the Hes5 promoter as Hes5^+^ cells, and those activating only the CAG promoter as CAG^+^ cells.

To confirm that the Hes5^+^ cells in the VZ were RGCs, we performed immunohistochemistry. We found that 48 h after electroporation the majority of the Hes5^+^ cells expressed the RGC marker Pax6 (Fig. 1*F*, arrows) but not the IP marker Tbr2 (Fig. 1*G*, arrowheads).

### Hes5^+^ RGCs mostly generate upper layer cortical neurons

**Figure 2.**
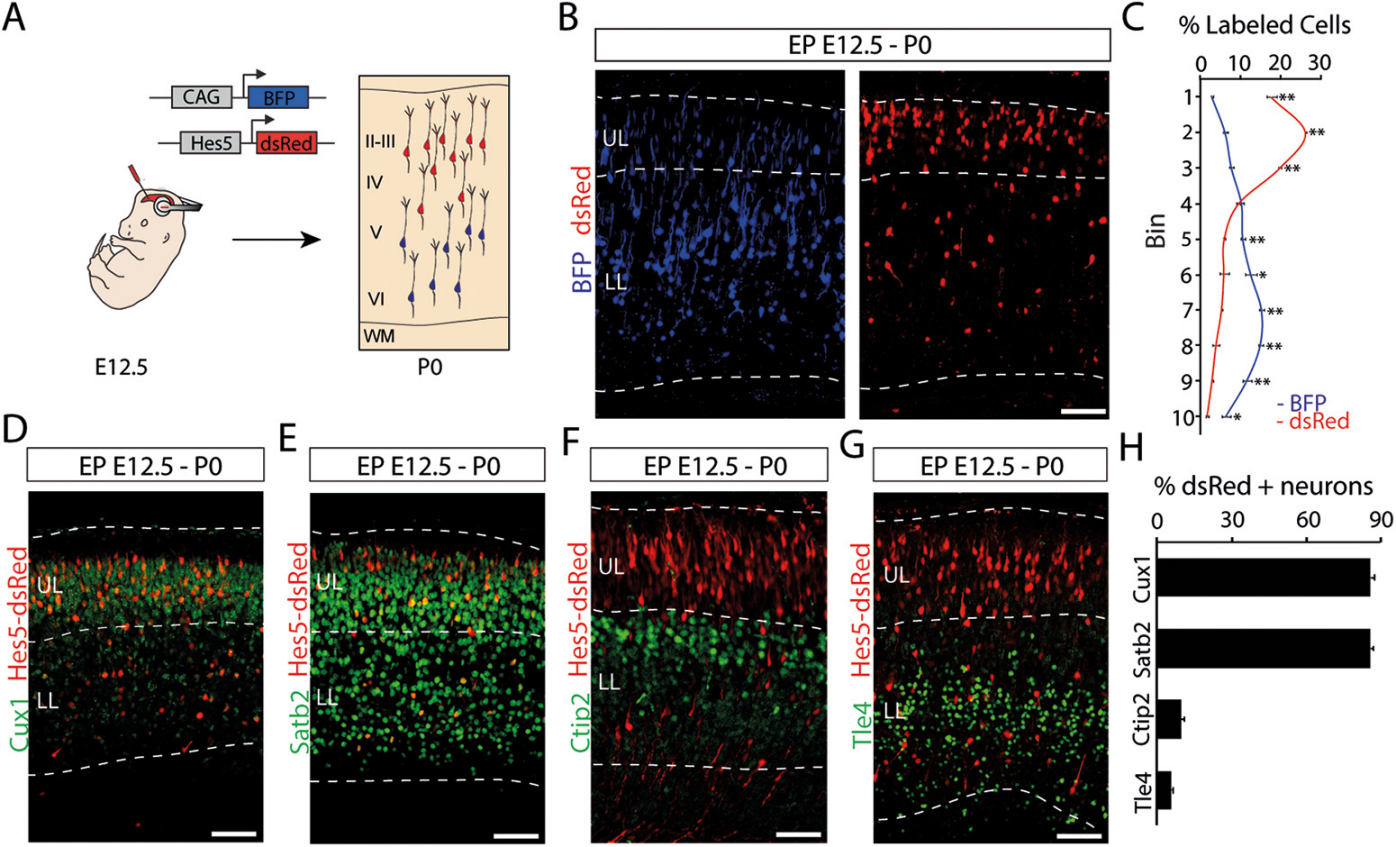
Laminar localization of neurons generated from early RGCs with delayed neurogenic potential. ***A***, Schematics of the electroporation strategy for laminar localization determination. ***B***, Coelectroporation of the CAG-BFP (blue) and Hes5-dsred (red) constructs at E12.5, with analysis at P0. ***C***, Quantification (mean ± SEM) of the distribution of electroporated cells in the CP at P0 from each subpopulation of progenitors. For quantification, the CP was divided in 10 equal-sized bins (enumerated 1–10 from basal to apical). *p < 0.02, **p < 0.0005. ***D–G***, Immunostaining (green) at P0 for Cux1 (***D***), Satb2 (***E***), Ctip2 (***F***), and Tle4 (***G***) of E12.5 Hes5p-dsRed (red) electroporated embryos. ***H***, Quantification (mean ± SEM) at P0 of Hes5p-dsRed electroporated cells expressing Cux1, Satb2, Ctip2, or Tle4. II–VI, Cortical layers 2–6; WM, white matter. Scale bars, 100 µm.

Hes5^+^ RGCs at early stages of corticogenesis appear to present delayed neurogenesis compared with other neurogenic progenitors at the same developmental stage. We next analyzed the distribution of electroporated cells at later stages after *in utero* electroporation to determine laminar localization of the neurons produced by Hes5^+^ cells (Hes5-dsRed) compared with CAG^+^ cells (CAG-BFP). We analyzed the laminar position of labeled cells 7 d after E12.5 coelectroporation of the two plasmids (Fig. 2*A*). CAG^+^ neurons were located within both the upper and lower parts of the developing CP, with greater accumulation in the lower part. On the other hand, Hes5^+^ neurons mostly accumulated in the upper part of the CP (CAG-BFP vs Hes5-dsRed: bin 1, *p* = 2.47 × 10^−5^; bin 2, *p* = 5.52 × 10^−8^; bin 3, *p* = 1.89 × 10^−6^; bin 5, *p* = 0.0002; bin 6, *p* = 0.0106; bin 7, *p* = 5.20 × 10^−6^; bin 8, *p* = 4.60 × 10^−5^; bin 9, *p* = 0.0002; bin 10, *p* = 0.0057; unpaired *t* test; *n* = 4; Fig. 2*B*,*C*). To determine the subtype of neurons that were Hes5^+^, we conducted immunohistochemistry using known markers for subtypes of neocortical neurons, namely Cux1, Satb2, Tle4, and Ctip2. Cux1 is a specific marker for upper layer neurons (Nieto et al., 2004), and the majority of Hes5^+^ cells located in the upper part of the CP also expressed Cux1 (85.72 ± 1.35%; Fig. 2*D*,*H*). Satb2 is a marker of corticocortical neurons, mostly located in upper layers but also present in some lower layer neurons (Alcamo et al., 2008; Britanova et al., 2008). Most of the Hes5^+^ cells were Satb2^+^ (85.74 ± 1.22%; Fig. 2*E*,*H*). Very few Hes5^+^ cells expressed Tle4, a marker for corticothalamic neurons in layer VI (Koop et al., 1996) or Ctip2, a marker for neurons in layers V and VI (Arlotta et al., 2005; Hes5^+^Tle4^+^/Hes5^+^, 9.71 ± 1.10%; Hes5^+^Ctip2^+^/Hes5^+^, 5.67 ± 0.98%; Fig. 2*F–H*). These results suggest that Hes5^+^ progenitors are involved in the generation of corticocortical projection neurons mostly located in upper layers.

**Figure 3.**
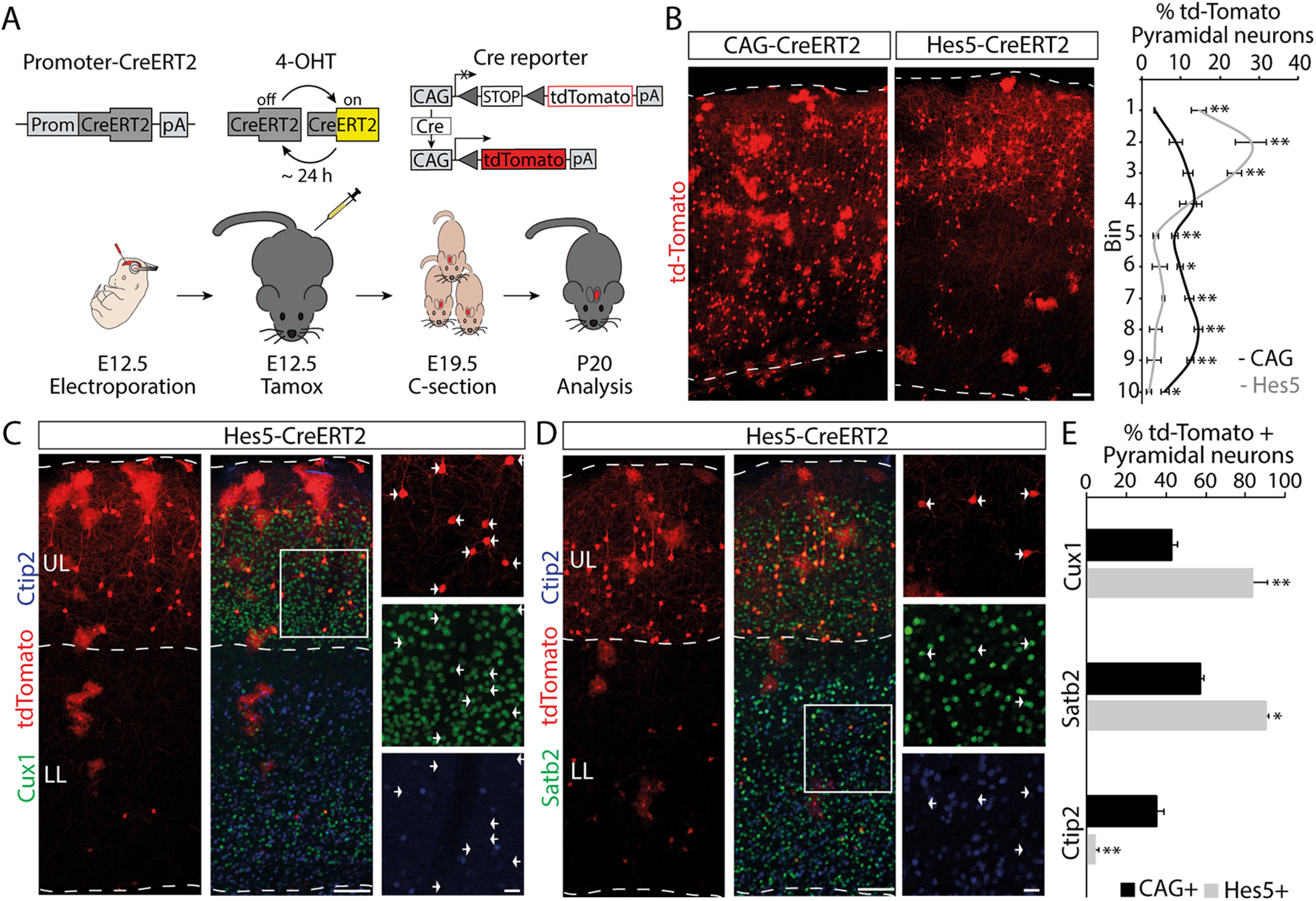
Lineage tracing of early RGCs activating Hes5 promoter. ***A***, Schematics of the linage-tracing strategy. Ai9 reporter embryos were electroporated at E12.5 with CAG-CreERT2 and Hes5-CreERT2 constructs. Pregnant dams were injected with 4-OHT right after electroporation. Pups were delivered at E19.5 by caesarean section. Brains were analyzed at P20. ***B***, Confocal images and quantification (mean ± SEM) of the distribution of tdTomato^+^ cells at P20. Note that most tdTomato^+^ neurons in the Hes5-CreERT2 electroporated brains were present in the upper layers, whereas CAG-CreERT2 brains show a more homogeneous distribution. For quantification, the CP was divided in 10 equal-sized bins (enumerated 1–10 from basal to apical). *p < 0.05, **p < 0.005 (Extended Data Fig. 3-1). ***C***, Double immunofluorescence for Cux1 (green) and Ctip2 (blue) on Hes5p-CreERT2 (red) electroporated brains showing magnification in the upper layers. Arrows point to examples of tdTomato^+^/Cux1^+^ cells. ***D***, Double immunofluorescence for Satb2 (green) and Ctip2 (blue) on Hes5-CreERT2 (red) electroporated brains showing magnification in the lower layers. Arrows point examples of tdTomato^+^/Satb2^+^ cells. ***E***, Quantification (mean ± SEM) on CAG-CreERT2 and Hes5-CreERT2 electroporated brains of recombined tdTomato^+^ cells expressing Cux1, Satb2, or Ctip2. *p < 0.01, **p < 0.0002. Abbreviations are as in Figure 2. Scale bars: ***B***, 100 µm; ***C***, ***D***, low magnification, 100 µm; ***C***, ***D***, boxed areas, 50 µm.

Hes5 is not expressed in differentiated neurons (Basak and Taylor, 2007), suggesting that the labeled neurons located in upper layers came from early Hes5^+^ RGCs. To formally test this possibility, we conducted lineage-tracing experiments. We generated a tamoxifen-inducible Cre-recombinase construct driven by the Hes5 promoter (Hes5-CreERT2) to conduct *in utero* electroporation at E12.5 in Ai9 reporter mice (Madisen et al., 2010). As a control, we used a general promoter (CAG-CreERT2). Since 4-OHT has a short half-life (Guenthner et al., 2013), we injected 4-OHT immediately after *in utero* electroporation to maximize recombination in RGCs taking up the plasmid, before they generated more differentiated cell types (Fig. 3*A*). We then analyzed the distribution of the neuronal offspring of electroporated RGCs at postnatal day 20 (P20). Following Hes5-CreERT2 electroporations and 4-OHT injection, there was a strong bias in the localization of td-Tomato^+^ pyramidal neurons toward upper layers when compared with CAG-CreERT2 electroporations (CAG-CreERT2 vs Hes5-CreERT2: bin 1, *p* = 0.0011; bin 2, *p* = 0.0043; bin 3, *p* = 0.002; bin 5, *p* = 0.0037; bin 6, *p* = 0.0417; bin 7, *p* = 0.001; bin 8, *p* = 0.0014; bin 9, *p* = 0.0037; bin 10, *p* = 0.0167; unpaired *t* test, *n* = 4; Fig. 3*B*). In addition to the neuronal offspring, we observed the presence of traced glial cells with astrocyte morphology (Fig. 3*B*) using both promoter fragments. The proportion of astrocytes versus pyramidal neurons was maintained in both conditions (percentage of td-Tomato astrocytes: CAG-CreERT2, 18.57 ± 3.28%; Hes5p-CreERT2, 15.72 ± 2.78%; *t*_(6)_ = 0.66, *p* = 0.53, unpaired *t* test; *n* = 4; Extended Data Fig. 3-1*A*). Similarly, no major differences in laminar distribution of astrocytes were found between the two conditions (CAG-CreERT2 vs Hes5-CreERT2: bin 4, *p* = 0.012, unpaired *t* test; *n* = 4; Extended Data Fig. 3-1*B*).

10.1523/JNEUROSCI.2905-20.2021.f3-1 Figure 3-1 Glial versus neural differentiation of RGCs activating Hes5 promoter. ***A***, Quantification (mean ± SEM) of the cell type of td-Tomato^+^ cells at P20. Ai9 reporter embryos were electroporated with Hes5-CreERT2 or CAG-CreERT2 plasmids. Morphological features were used to distinguish pyramidal cells and astrocytes. ***B***, Quantification (mean ± SEM) of the distribution of td-Tomato^+^ astrocytes within the CP at P20. Ai9 reporter embryos were electroporated with Hes5-CreERT2 or CAG-CreERT2 plasmids. For quantification, the CP was divided into 10 equal-sized bins (enumerated 1–10 from basal to apical). *p < 0.05. Download Figure 3-1, TIF file.

Most of the td-Tomato^+^ neurons in brains electroporated with Hes5-CreERT2 expressed the upper layer marker Cux1 (83.96 ± 7.30%; Fig. 3*C*,*E*) and the corticocortical marker Satb2 (90.81 ± 0.89%; Fig. 3*D*,*E*), even when located in lower layers (Fig. 3*D*). Very few of them expressed Ctip2 (3.79 ± 1.48%), which is mostly expressed by corticofugal neurons and some interneurons (Arlotta et al., 2005; Fig. 3*C–E*). In contrast, td-Tomato^+^ neurons in brains electroporated with CAG-CreERT2 comprised higher percentages of Ctip2^+^ neurons (37.97 ± 7.22%; *t*_(4)_ = 2.13, *p* = 0.0097, unpaired *t* test; *n* = 3) and reduced the numbers of Cux1^+^ and Satb2^+^ recombined neurons (Cux1^+^, 38.70 ± 1.98%; *t*_(4)_ = −5.98; *p* = 0.0039; Satb2^+^: 56.96 ± 2.34%; *t*_(4)_ = −13.51; *p* = 0.00017; unpaired *t* test; *n* = 3; Fig. 3*E*).

### Single-cell RNA sequencing of early Hes5^+^ RGCs reveals a molecular signature of cortical progenitors with delayed neurogenic potential

**Figure 4.**
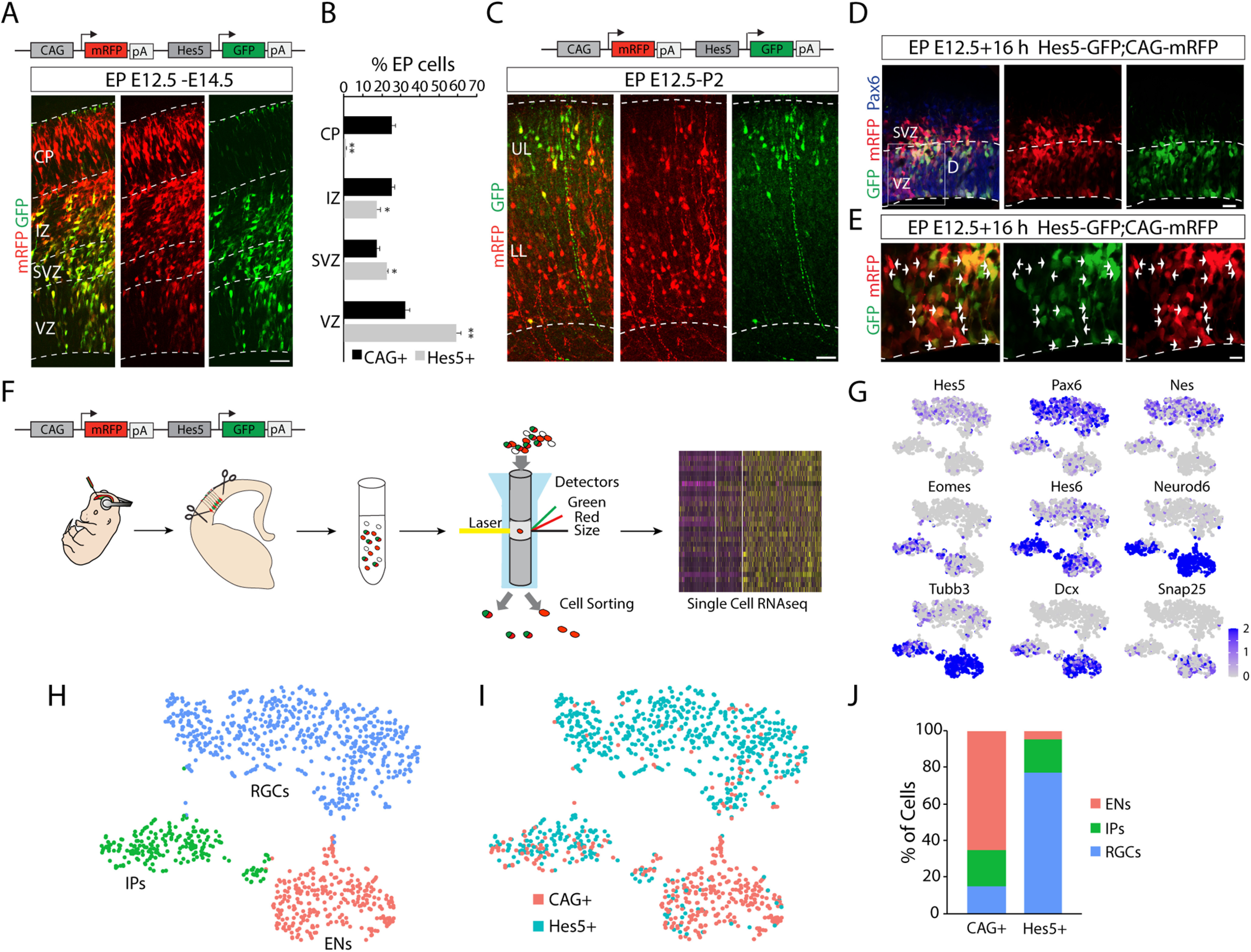
Single-cell sorting, sequencing and scRNA-seq identification analysis. ***A***, *In utero* electroporation experiment at E12.5 using the dual reporter plasmid CAG-mRFP;Hes5-GFP analyzed at E14.5. Note the differential distribution of the cells expressing mRFP (red) and the cells expressing GFP (green). ***B***, Quantification of the data in ***A*** (mean ± SEM) showing the different proportion of CAG^+^ and Hes5^+^ cells in the VZ, SVZ, IZ, and CP at E14.5. *p < 0.05; **p < 0.0002. ***C***, *In utero* electroporation experiment at E12.5 using the dual reporter plasmid CAG-mRFP;Hes5-GFP analyzed at P2. Note the differential laminar localization of the projection neurons activating the Hes5 promoter. ***D***, Immunostaining for Pax6 (blue) on EP E12.5 electroporated embryos with the dual promotor plasmid CAG-mRFP;Hes5p-GFP analyzed 16 h after electroporation. ***E***, Higher-magnification view of boxed area in ***D***. Arrowheads point mRFP^+^/GFP^+^ cells, and arrows point to mRFP^+^/GFP^–^ cells. ***F***, Schematics of scRNA-seq strategy. The electroporated area was isolated and cells were individually separated by FACS 16 h after electroporation. Isolated cells were sequenced and analyzed using bioinformatics tools. ***G***, t-SNE visualizations of the sorted cells displaying gene expression (gray to dark blue color gradient) of cell type-specific markers for RGCs (Hes5, Pax6, Nes), IPs (Eomes and Hes6), and early neurons (Neurod6, Tubb3, Dcx, and Snap25). ***G***, t-SNE visualizations of the sorted cells displaying gene expression (gray to dark blue color gradient) of cell type-specific markers for RGCs (Hes5, Pax6, Nes), IPs (Eomes and Hes6), and early neurons (Neurod6, Tubb3, Dcx, and Snap25). ***H***, t-SNE visualizations of the scRNA-seq dataset according to expression of the cell type-specific markers (shown in ***G***) and particular cell populations identified (blue, RGCs; green, IPs; red, early neurons). ***I***, t-SNE visualizations of the scRNA-seq dataset exhibiting CAG^+^ cell group (mRFP^+^; red) versus Hes5^+^ cell group (mRFP^+^, GFP^+^; green). ***J***, Proportion analysis of CAG^+^ and Hes5^+^ cells in the three groups of cell types. Hes5^+^ cells accounted for the majority of cells with an RGC transcriptional signature, whereas CAG^+^ cells constituted the vast majority of ENs. Scale bars: ***A***, ***C***, ***D***, 50 µm; ***E***, 25 µm.

The previous results could suggest the existence of phenotypic heterogeneity among RGCs and are compatible with the presence of two populations of RGCs at these particular ages: one being early neurogenic and other remaining longer as RGCs before generating neurons. To search for molecular differences between these RGCs, we designed an experiment to isolate neocortical progenitor cells and characterize them by single-cell scRNA-seq. For this purpose, we generated a dual promoter plasmid containing two fluorescent proteins under the control of the following two different promoters: the CAG promoter driving mRFP and the Hes5 promoter driving GFP. This strategy ensures that both reporter constructs are present within each electroporated cell. Validation of the construct by *in utero* electroporation at E12.5 confirmed a similar distribution of labeled cells at different time points after IUE, as we described with the individual plasmids (Figs. 1, 2, 4*A–C*). Two days after electroporation, reduced numbers of Hes5^+^ cells were located in the CP compared with CAG^+^ cells (CAG^+^, 25.28 ± 1.84; Hes5^+^, 0.90 ± 0.36; *t*_(6)_ = 12.95, *p* = 1.29 × 10^−5^, unpaired *t* test; *n* = 4; Fig. 4*A*,*B*). Conversely, the proportion of Hes5^+^ cells present in the VZ was significantly increased compared with the proportion of CAG^+^ cells (CAG^+^, 32.21 ± 2.42; Hes5^+^, 59.25 ± 2.18; *t*_(6)_ = −8.28, *p* = 0.0002, unpaired *t* test; *n* = 4; Fig. 4*B*). Small differences in the distribution of labeled cells were also observed in the SVZ (CAG^+^, 17.44 ± 1.46; Hes5^+^, 22.40 ± 0.84; *t*_(6)_ = −2.93, *p* = 0.03, unpaired *t* test; *n* = 4; Fig. 4*B*) and in the IZ (CAG^+^, 22.05 ± 1.61; Hes5^+^, 17.44 ± 1.73; *t*_(6)_ = 3.22, *p* = 0.02, unpaired *t* test; *n* = 4; Fig. 4*B*). Analysis at later ages (P2) confirmed that Hes5^+^ cells were preferentially located within upper layers after E12.5 electroporation using the dual vector (Fig. 4*C*).

To conduct the scRNA-seq experiments, we adjusted the timing between the surgery for *in utero* electroporation at E12.5 and cell isolation to optimize fluorescence intensity of cells largely confined to the proliferative zone of the neocortex (Fig. 4*D*). The optimal time point for cell sorting was 16 h after *in utero* electroporation, displaying substantial numbers of Pax6^+^ RGCs (Fig. 4*D*) expressing either only mRFP (Fig. 4*E*, arrows) or both fluorescent proteins (Fig. 4*E*, arrowheads). Labeled brains were dissected, and the electroporated areas of the neocortex were isolated, dissociated into single cells, and sorted using fluorescence-activated cell sorting (FACS) to separate CAG^+^ cells (only CAG promoter active, mRFP^+^GFP^–^ cells) and Hes5^+^ cells (both promoters active, mRFP^+^GFP^+^ cells; Fig. 4*F*). Viable sorted cells of both types collected individually were lysed, and libraries were generated using a modified Smart-seq2 protocol for subsequent scRNA-seq, as described previously (Chevée et al., 2018).

Data were analyzed using the Seurat R package (Butler et al., 2018). A total of 1115 cells passed our quality control test. From those cells, we obtained 731 CAG^+^/Hes5^+^ (mRFP^+^ and GFP^+^) and of 384 CAG^+^ cells (mRFP^+^ but GFP^–^). By using cell type-specific markers, sequenced cells could be assigned to the following three main cell types of the developing neocortex: RGCs (Pax6, Vim, Hes5; Schnitzer et al., 1981; Götz et al., 1998; Hatakeyama et al., 2004); IPs (Eomes, Hes6; Englund et al., 2005; Kawaguchi et al., 2008); and early neurons (ENs; Neurod6, Tubb3, Snap25; Caccamo et al., 1989; Oyler et al., 1989; Goebbels et al., 2006; Fig. 4*G*,*H*).

We next compared the distribution of Hes5^+^ (mRFP^+^GFP^+^) and CAG^+^ (mRFP^+^GFP^–^) cells between the main cell-type clusters (Fig. 4*I*). While the proportion of IPs was similar for both groups (17.92% of Hes5^+^ cells and 19.79% of CAG^+^ cells), most of the CAG^+^ cells were categorized as ENs (65.37%). Only a small percentage of the Hes5^+^ cells were neurons (4.79%; Fig. 4*J*), suggesting that CAG^+^ RGCs differentiate more readily at the time of electroporation (E12.5). Conversely, Hes5^+^ cells largely had the transcriptional signature of RGCs (77.29%), while only a small fraction of CAG^+^ cells displayed RGC characteristics (14.84%; Fig. 4*J*).

**Figure 5.**
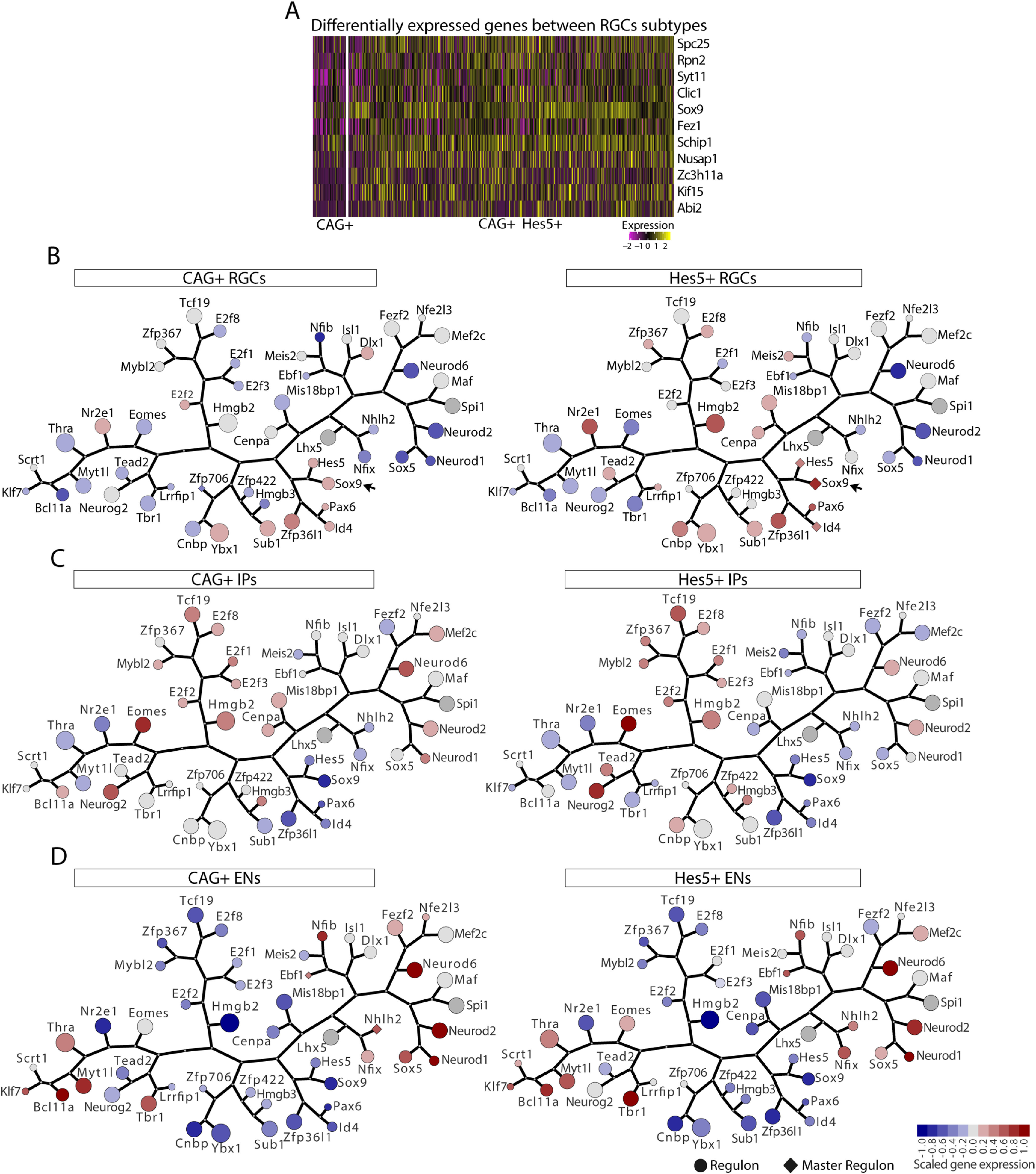
Transcriptional analysis reveals differentially expressed genes and master regulons. ***A***, Heatmap of differentially expressed genes (rows) in RGCs belonging to one of the subgroups comparisons (Hes5^+^ vs CAG^+^; columns). Gene expression levels are colored ranging from twofold downregulation (pink) to twofold upregulation (yellow), according to the color key. Only genes with an adjusted *p* value < 0.05 and log-fold change > 0.3 are shown. ***B–D***, Transcriptional regulatory network of scRNA-seq data displaying expression levels of each regulon in RGCs (***B***), IPs (***C***), and ENs (***D***) in CAG^+^ and Hes5^+^ cells. Circles are regulons and diamonds master regulons. Circle/diamond size is related to number of genes orchestrated by its transcription factor. Gene expression levels are colored according to average gene expression. The tree network represents correlation among regulons. Note that RGCs transcriptional regulatory network exhibits Sox9 as a master regulator gene specific of Hes5^+^ RGCs.

Next, we set out to identify gene expression signatures that could distinguish Hes5^+^ from CAG^+^ RGCs (Fig. 5). We first investigated DEGs in these two populations using a Wilcoxon rank-sum test (Fig. 5*A*). We identified 11 genes whose expression was significantly upregulated in the subpopulation of Hes5^+^ compared with CAG^+^ RGCs (adjusted *p* < 0.05; average log2FC > 0.3; Fig. 5*A*). Among these DEGs, we identified Sox9 as a candidate transcription factor that could play a key role in the regulation of the fate of neural stem cells (NSCs; Stolt et al., 2003; Vong et al., 2015; Kaplan et al., 2017). To investigate whether Sox9 could be an important regulator of gene expression in Hes5^+^ RGCs, we generated a transcriptional regulatory network and searched for master regulators (Margolin et al., 2006; Fletcher et al., 2013; Castro et al., 2016) using previously published scRNA-seq data at the same age as our analysis (E13; Yuzwa et al., 2017; a total of 1944 cells and 16,378 genes). A transcriptional regulatory network consists of a collection of regulated target genes and TFs, in which the set of genes controlled by a given TF forms a regulon (Margolin et al., 2006; Fletcher et al., 2013; Castro et al., 2016). We found a GRN containing 49 regulons differently expressed in RGCs, Ips, and ENs (Fig. 5*B–D*). We also observed that the regulatory network of RGCs could be distinguished from those of IPs and ENs by higher expression of Hes5, Sox9, Pax6, Id4, and Zfp36l1 (Fig. 5*B*–*D*). Moreover, the Sox9 and Hes5 regulons were next to each other in the GRN, indicating that these two transcription factors share common target genes. We next used master regulator analysis (Carro et al., 2010; Castro et al., 2016) to analyze the enrichment of DEGs identified in Hes5^+^ RGCs compared with CAG^+^ RGCs (Fig. 5*A*) in our GRN. We observed that those DEGs were significantly enriched in Sox9, Hes5, and Id4 regulons (Fig. 5*B*), suggesting that these TFs are master regulators of this gene set in Hes5^+^ RGCs. Altogether, these findings suggest that Sox9 could be an important fate determinant in Hes5^+^ RGCs.

**Figure 6.**
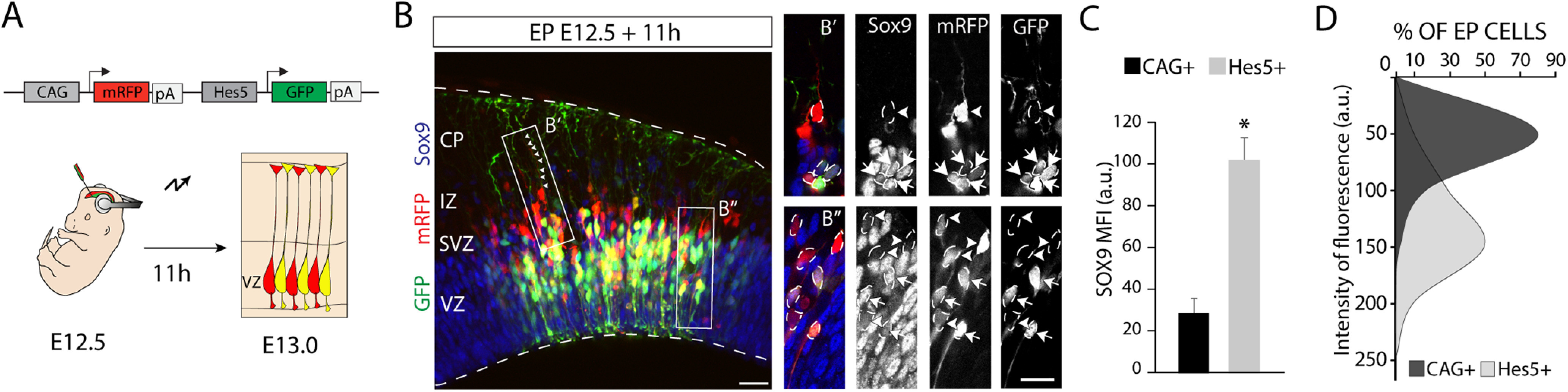
RCGs activating the Hes5 promoter display increased levels of Sox9 protein. ***A***, Schematic illustration of the electroporation strategy to verify Sox9 expression differences indicated by scRNA-seq analysis. E12.5 embryos were electroporated with the dual promotor plasmid CAG-mRFP;Hes5-GFP (red and green, respectively), analyzed 11 h after electroporation. ***B***, Immunostaining for Sox9 (blue) after CAG-mRFP;Hes5-GFP electroporation (red; green). ***B′***, ***B′′***, Higher-magnification of boxed areas in ***B***. Examples of CAG^+^ RGCs (arrowheads) and Hes5^+^ RGCs (arrows). Note that Hes5^+^ RGCs show higher levels of Sox9 immunostaining. Small arrowheads point to the basal process of an RGC. ***C***, Quantification (mean ± SEM) of the MFI of Sox9 in CAG^+^ versus Hes5^+^ RGCs. MFI in a scale of 0–255 a.u. *p < 0.0015. ***D***, Distribution of the Sox9 intensity of fluorescence (in arbitrary units) for CAG^+^ and Hes5^+^ RGCs. Note the differences in the percentages of each subtype of cells for the different intensities of fluorescence. Scale bars: ***B***, 50 µm; ***B′***, ***B′′***, 20 µm.

To verify differences in Sox9 protein expression between the two RGCs subtypes, we performed *in utero* electroporation using the dual promoter plasmid and conducted Sox9 immunohistochemistry 11 h after the surgery (Fig. 6*A*). Quantitative analysis of Sox9 staining in Hes5^+^ (Fig. 6*B*, arrows) and CAG^+^ RGCs (Fig. 6*B*, arrowheads) confirmed statistically significant differences in Sox9 expression levels between those RGCs, as shown by the analysis of the mean fluorescence intensity (CAG^+^, 29.15 ± 10.58 a.u.; Hes5^+^, 101.99 ± 10.58 a.u.; *t*_(6)_ = −5.88, *p* = 0.001, unpaired *t* test; *n* = 4; Fig. 6*C*) and the evident differential distribution of Sox9 fluorescence intensity between CAG^+^ and Hes5^+^ RGCs (Fig. 6*D*).

### Sox9 overexpression in RGCs affects early neuronal differentiation

**Figure 7.**
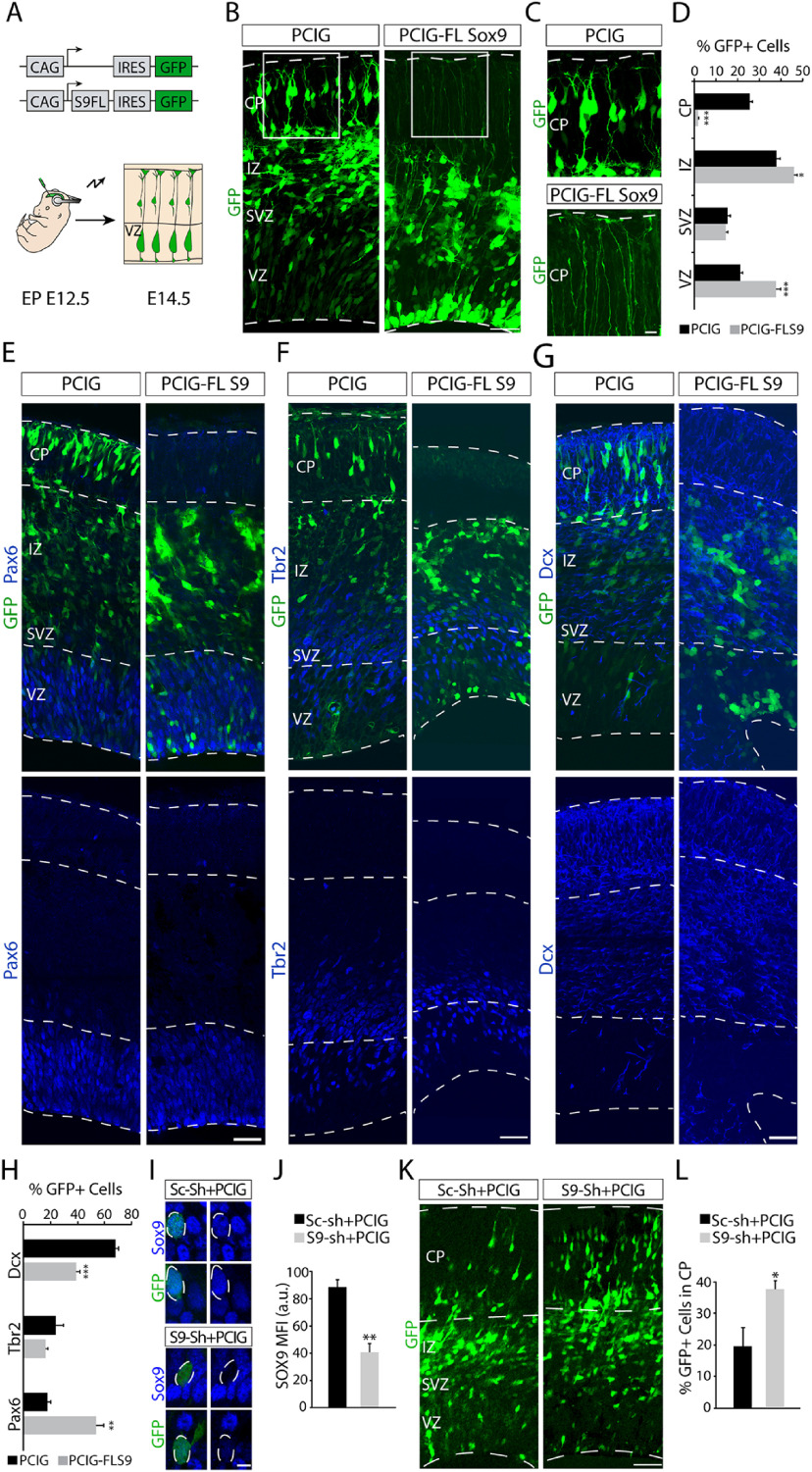
Sox9 overexpression affects neurogenic behavior of RGCs. ***A***, Schematics of the electroporation strategy. E12.5 embryos were electroporated with the PCIG-FL Sox9 plasmid to overexpress Sox9, and its empty version, the PCIG plasmid (GFP only), was used as a control. Brains were analyzed 48 h after electroporation. ***B***, Confocal images of the neocortex of an E14.5 embryo after PCIG or PCIG-FL Sox9 electroporation (green). Note that Sox9 overexpression caused a lack of neurons in the CP and cellular accumulation in the VZ compared with the control condition. ***C***, Higher-magnification image of the CP showing the end feet of RGCs. Note that Sox9 overexpression did not affect the RGC scaffold. ***D***, Quantification (mean ± SEM) of the distribution of targeted cells within the developing neocortex (E14.5). *p < 0.05; ***p < 0.0005. ***E–G***, Immunochemistry at E14.5 brains for Pax6 (***E***), Tbr2 (***F***), and Dcx (***G***; blue) on E12.5 PCIG or PCIG-FL Sox9 (green) electroporated embryos. ***H***, Quantification (mean ± SEM) at E14.5 of PCIG and PCIG-FL Sox9 electroporated cells expressing Pax6, Tbr2, or Dcx. **p < 0.005; ***p < 0.0005. ***I***, Immunostaining at the E14.5 VZ for Sox9 (blue) after E12.5 electroporation (green) with sc-shRNA^+^PCIG plasmids, used as a control, and Sox9-shRNA^+^PCIG plasmids, used to downregulate Sox9. Note that in the knock-down condition Sox9 intensity was successfully reduced in electroporated cells in the VZ. ***J***, Quantification (mean ± SEM) of the mean fluorescence intensity of Sox9 in electroporated cells in the VZ at E14.5. **p < 0.005. ***K***, Confocal image of E14.5 brains after E12.5 electroporation with sc-shRNA^+^PCIG plasmids and Sox9^–^shRNA^+^PCIG plasmids. ***L***, Quantification (mean ± SEM) of the percentage of electroporated cells located in the CP at E14.5. *p < 0.05. Scale bars: ***B***, 100 µm; ***C***, 20 µm; ***E–G***, ***K***, 50 µm; ***I***, 10 µm.

Sox9 is required for stem cell maintenance in different tissues (Scott et al., 2010; Roche et al., 2015) as well as for suppression of cell differentiation (Kadaja et al., 2014). In the CNS, overexpression of Sox9 in the adult SVZ (aSVZ) abolishes the production of neurons, whereas Sox9 knockdown increases neurogenesis and reduces gliogenesis (Cheng et al., 2009). Because Sox9 is a putative regulator enriched in the Hes5^+^ RGCs, we predicted that increased levels of Sox9 observed in those progenitors may affect the proportion of differentiative divisions. To test this hypothesis, we overexpressed Sox9 in RGCs at E12.5 using a general promoter driving Sox9 and GFP (PCIG-FL Sox9) and analyzed possible changes in differentiation at E14.5 (Fig. 7*A*). Sox9 overexpression affected the distribution of electroporated cells with reduced invasion of the CP and a greater accumulation of labeled cells in the VZ (PCIG vs PCIG-FL S9: VZ, *p* = 0.00049; IZ, *p* = 0.011; CP, *p* = 1.80 × 10^−6^; unpaired *t* test; *n* = 4; Fig. 7*B–D*). Overexpression of Sox9 did not noticeably affect the RGCs scaffold, as revealed by the visualization of their end feet (Fig. 7*C*). Detailed analysis of the cells accumulated in the VZ using markers for RGCs (Pax6), IPs (Tbr2), and postmitotic neurons (Dcx) revealed that the Sox9-overexpressing cells were RGCs (Fig. 7*E*–*G*). Moreover, quantifications of molecular markers for progenitors and differentiating neurons showed a statistically significant increase in the number of Pax6^+^ cells on Sox9 overexpression (PCIG, 18.02 ± 1.89%; PCIG-FL Sox9, 53.42 ± 5.90%; *t*_(6)_ = −5.72, *p* = 0.0012, unpaired *t* test; *n* = 4; Fig. 7*E*,*H*) and a concomitant decrease in the number of Dcx^+^ early neurons (PCIG, 68.07 ± 2.17%; PCIG-FL Sox9, 38.85 ± 2.77%; *t*_(6)_ = 8.31, *p* = 0.00,016, unpaired *t* test; *n* = 4; Fig. 7*G*,*H*), suggesting reduced differentiative divisions on Sox9 overexpression.

Because Sox9 overexpression seems to delay neuron production during early stages of corticogenesis, we determined the extent to which Sox9 downregulation in the early cortical VZ increases the neurogenic behavior of RGCs, as described in the aSVZ (Cheng et al., 2009). To this end, we performed short-time *in utero* electroporation from E12.5 to E14.5 using previously validated Sox9 or scrambled shRNA together with the PCIG vector (Saharia et al., 2008; Guo et al., 2012). The analysis of the expression of Sox9 in electroporated samples confirmed the reduction in Sox9 levels in RGCs targeted with the Sox9-shRNA in comparation with the control situation (sc-shRNA, 88.33 ± 5.46 a.u.; S9-shRNA, 40.72 ± 6.20 a.u.; *t*_(4)_ = 5.76, *p* = 0.0045, unpaired *t* test; *n* = 3; Fig. 7*I*,*J*). These Sox9 KD experiments showed an increase in the number of targeted neurons occupying the cortical plate (sc-shRNA, 19.62 ± 6.10%; S9-shRNA, 37.75 ± 2.75%; *t*_(6)_ = −2.71, *p* = 0.035, unpaired *t* test; *n* = 4; Fig. 7*K*,*L*), suggesting that reductions in Sox9 expression favors neurogenic divisions.

### Sox9 overexpression affects RGCs cell cycle duration

**Figure 8.**
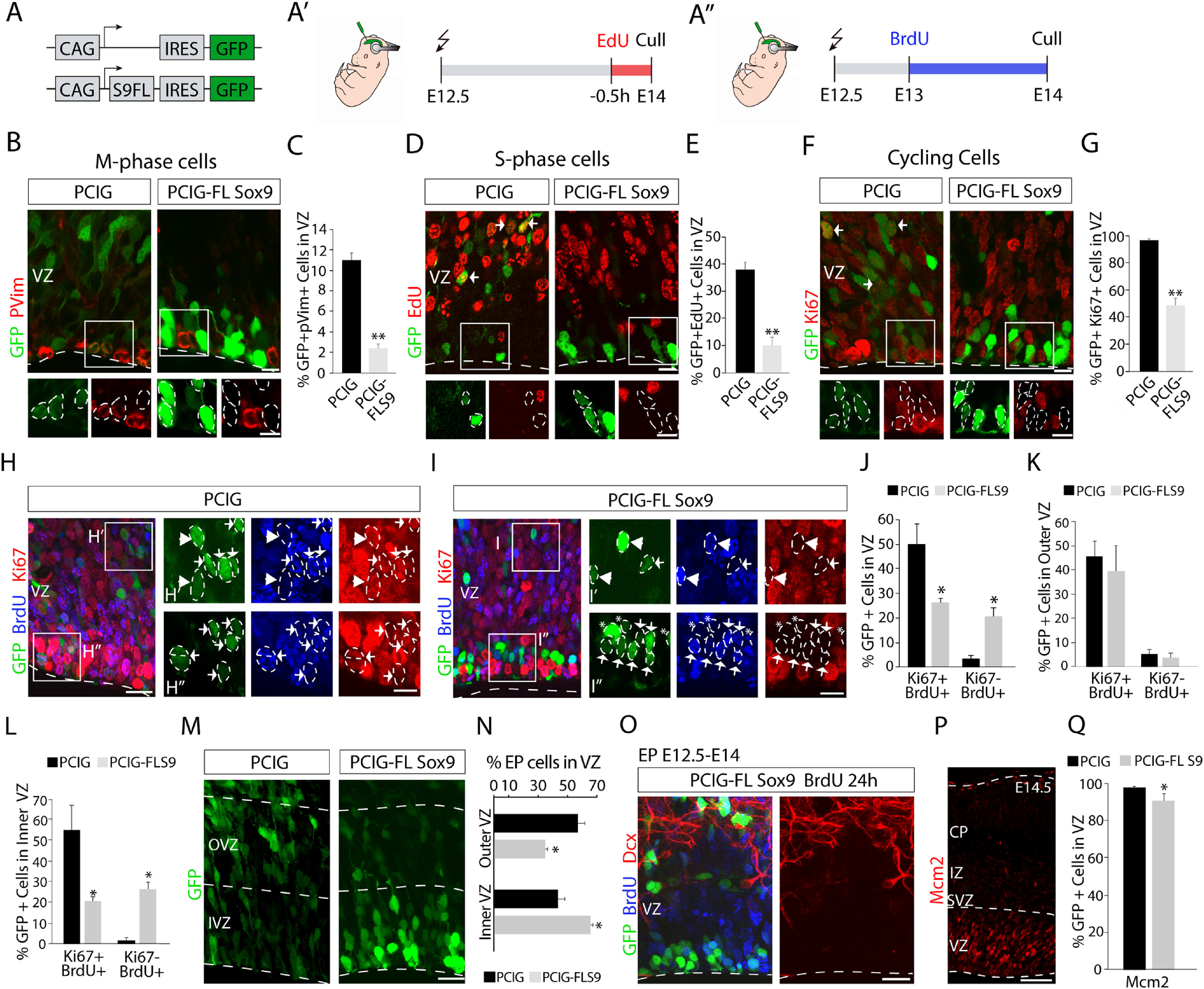
Analysis of cell cycle markers reveal alterations in RGCs overexpressing Sox9. ***A***, Schematics of the electroporation strategy. E12.5 electroporated embryos with the PCIG or PCIG-FL Sox9 plasmids were analyzed 36 h postelectroporation (E14.0). ***A′***, ***A′′***, Electroporation was combined with the injection of nucleoside analogs. ***B***, Immunochemistry for M-phase marker pVim (red) 36 h postelectroporation (green). Note that most cells present on the surface of the ventricle in the Sox9 overexpression condition are not in M phase. ***C***, Quantification (mean ± SEM) of electroporated cells expressing pVim at E14.0. **p < 0.0002. ***D***, EdU staining (red) in electroporated brains (green) after a single pulse of EdU, 0.5 h before dissection, as illustrated in ***A′***. Arrows point to examples of GFP^+^EdU^+^ cells. ***E***, Quantification (mean ± SEM) at E14.0 of electroporated cells labeled with EdU. **p < 0.0002. ***F***, Immunochemistry for proliferation marker Ki67 (red) 36 h postelectroporation (green). ***G***, Quantification (mean ± SEM) of electroporated cells expressing Ki67 at E14.0. **p < 0.0002. ***H***, ***I***, Costaining for BrdU (blue) and proliferation marker Ki67 (red) 36 h after electroporation (green) with PCIG (***H***) or PCIG-FL Sox9 plasmid (***I***). As illustrated in ***A′′***, pregnant dams received a single pulse of BrdU 12 h postelectroporation (E13.0), and embryos were dissected 24 h post-BrdU injection (E14.0). ***H′***, ***H′′***, Higher-magnification view of boxed areas in ***H***. ***I′***, ***I′′***, Higher-magnification view of boxed areas in ***I***. Arrows point to examples of EP cells stained for Ki67 and BrdU, arrowheads point to EP cells stained only for BrdU, and asterisks point to EP cells stained only for GFP. ***J***, Quantification (mean ± SEM) of EP cells that are Ki67^+^BrdU^+^ or Ki67^–^BrdU^+^ in the VZ at E14.0. *p < 0.05. ***K***, ***L***, Quantification (mean ± SEM) of the distribution within the VZ of EP cells that are Ki67^+^BrdU^+^ or Ki67^–^BrdU^+^ at E14.0. *p < 0.05. For these quantifications, the VZ was divided in two equal-sized regions: the outer (***K***) and the inner (***L***) VZ. *p < 0.05. Note that the differences in the percentages of EP cells Ki67^+^BrdU^+^ between the control and overexpression condition shown in ***J*** are mainly caused by the differences in the IVZ shown in ***L***. ***M***, Confocal image of the VZ at E14.0 after E12.5 PCIG or PCIG-FL Sox9 electroporation. ***N***, Quantification (mean ± SEM) of the distribution within the VZ of EP cells with PCIG and PCIG-FL Sox9. *p < 0.05. ***O***, Costaining for BrdU (blue) and Dcx (red) 36 h postelectroporation (green). Note that EP cells BrdU^+^ in the IVZ are not Dcx^+^. ***P***, Full cortex view of the staining for the proliferation marker Mcm2 at E14.0. ***Q***, Quantification (mean ± SEM) of EP cells that express Mcm2 in the VZ at E14.0. *p < 0.05. Scale bars: ***B***, ***D***, ***F***, low magnification, 15 μm; ***B***, ***D***, ***F***, boxed areas, 10 μm; ***H***, ***I***, ***M***, ***O***, 20 μm; ***H***', ***H***'', ***I′***, ***I′′***, 10 μm; ***P***, 100 μm.

The imbalance in the neuronal production seen on Sox9 perturbation could be caused by alterations in the cell cycle of RGCs. To test this hypothesis, we performed a cell cycle analysis comparing the cortical VZ of embryos electroporated at E12.5 with control plasmid (PCIG) or the Sox9 overexpression plasmid (PCIG-FL Sox9) 36 h after the surgery (E14.0; Fig. 8*A*–*A′′*). We first evaluated the proportion of GFP^+^ cells in the VZ that were in different cell cycle phases using immunohistochemistry for known cell cycle markers including phosphorylated vimentin (pVim) for M-phase (Kamei et al., 1998; Noctor et al., 2002) and Ki67, which labels cycling cells in all phases of the cell cycle (Gerdes et al., 1983). Moreover, we applied short time pulses of the thymidine analog EdU (30 min) to evaluate the proportion of cells in S phase (Buck et al., 2008; Fig. 8*A′*).

Upon Sox9 overexpression, we observed the following significant reductions in the number of positive cells for pVim, EdU, and Ki67: pVim^+^ (PCIG, 11.02 ± 0.67%; PCIG-FL Sox9, 2.40 ± 0.47%; *t*_(6)_ = 10.52, *p* = 4.34 × 10^−5^, unpaired *t* test; *n* = 4, Fig. 8*B*,*C*); EdU^+^ (PCIG, 37.95 ± 2.70%; PCIG-FL Sox9, 10.52 ± 3.01%; *t*_(6)_ = 6.79, *p* = 0.0005, unpaired *t* test; *n* = 4; Fig. 8*D*,*E*); and Ki67^+^ (PCIG, 96.03 ± 1.06%; PCIG-FL Sox9, 48.61 ± 5.29%; *t*_(6)_ = 8.79, *p* = 0.00,012, unpaired *t* test; *n* = 4; Fig. 8*F*,*G*). These data together suggest that Sox9 overexpression significantly affected the cell cycle in the electroporated cells.

To investigate possible differences in cell cycle exit, we electroporated mice at E12.5 to overexpress Sox9. We then injected BrdU 12 h after surgery (E13.0) to label cells in S phase and analyze electroporated brains 24 h after the injection (E14.0; Fig. 8*A′′*). Double immunohistochemistry to detect BrdU and Ki67 staining in GFP^+^ cells in the VZ (Fig. 8*H*,*I*) revealed a dramatic reduction in the number of Ki67^+^BrdU^+^ cycling cells in the overexpression condition, coupled with an increase in the proportion of Ki67^–^BrdU^+^ cells (percentage of GFP^+^Ki67^+^BrdU^+^ cells in VZ: PCIG, 49.97 ± 13.74%; PCIG-FL Sox9, 26.17 ± 6.65%; *t*_(4)_ = 2.88, *p* = 0.045; percentage of GFP^+^Ki67^–^BrdU^+^ cells in VZ: PCIG, 3.65 ± 1.40%; PCIG-FL Sox9, 20.45 ± 5.72%; *t*_(4)_ = −4.29, *p* = 0.013, unpaired *t* test; *n* = 3; Fig. 8*J*). These differences were mainly detected in the IVZ (percentage of GFP^+^Ki67^+^BrdU^+^, PCIG vs PCIG-FL Sox9: IVZ, *p* = 0.047; OVZ, *p* = 0.66; percentage of GFP^+^Ki67^–^BrdU^+^, PCIG vs PCIG-FL Sox9: IVZ, *p* = 0.0065; OVZ, *p* = 0.64; *n* = 4; Fig. 8*K*,*L*), where many of the targeted cells accumulate on Sox9 overexpression (PCIG, 43.27 ± 4.75%; PCIG-FL Sox9, 65.37 ± 1.63%; *t*_(6)_ = −4.40, *p* = 0.0046, unpaired *t* test; *n* = 4; Fig. 8*M*,*N*). These results might suggest that part of the cycling cells incorporating BrdU at E13.0 exited the cell cycle but remained in the VZ. However, these GFP^+^ cells did not express markers for differentiated neurons like Dcx (Figs. 7*G*, 8*O*) or IP markers like Tbr2 (Fig. 7F). Instead, they maintained the expression of the RGC marker Pax6 (Fig. 7*E*), suggesting that the cells did not differentiate but still maintained progenitor properties.

**Figure 9.**
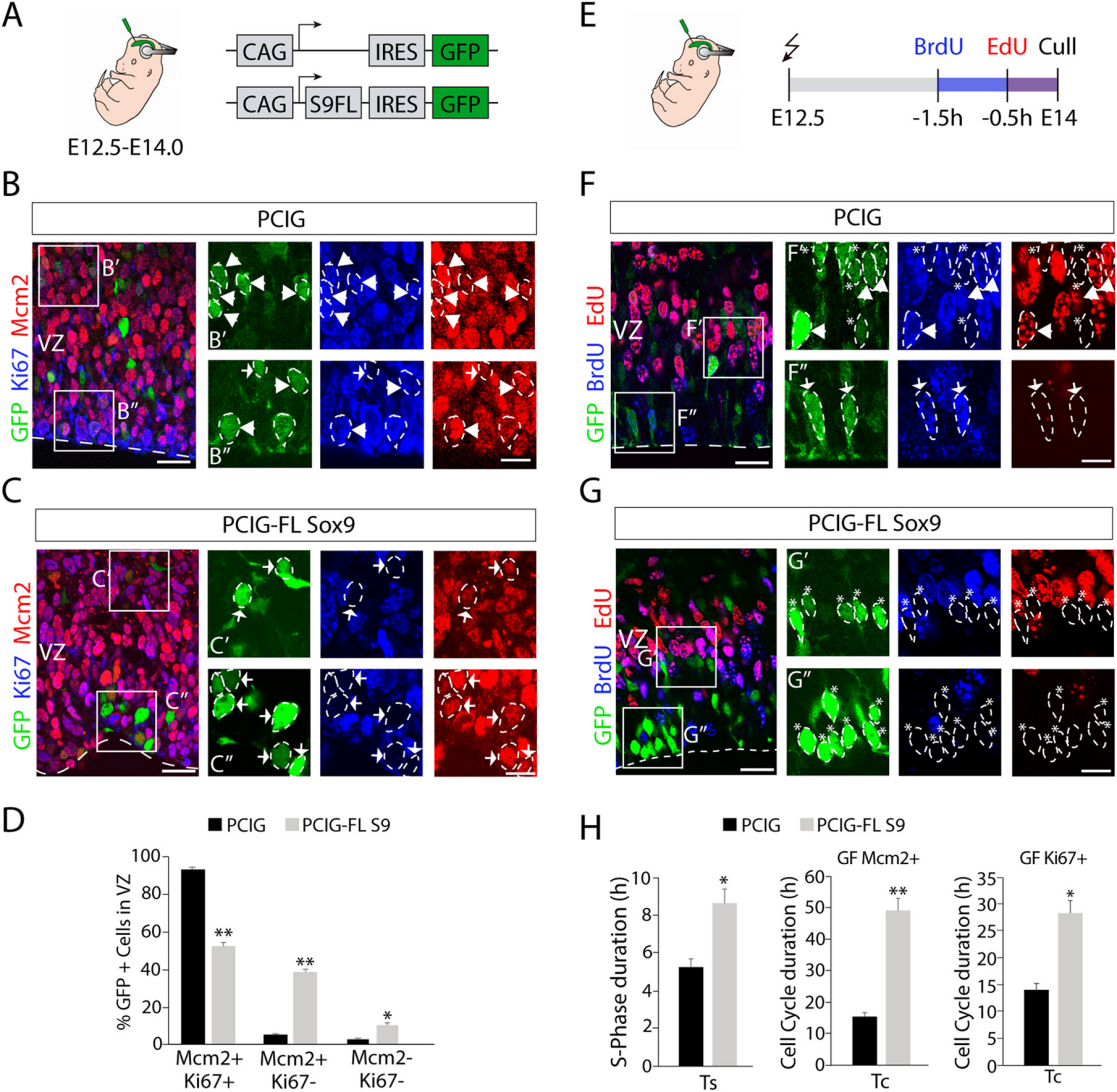
Sox9 overexpression affects RGCs cell cycle duration. ***A***, Schematics of the electroporation strategy. E12.5 electroporated embryos with the PCIG or PCIG-FL Sox9 plasmids were analyzed at E14.0. ***B***, ***C***, Costaining for proliferation markers Mcm2 (red) and Ki67 (blue) 36 h after electroporation (green) with PCIG (***B***) or PCIG-FL Sox9 (***C***) plasmids. ***B′***, ***B′′***, Higher-magnification views of boxed areas in ***B***. ***C′***, ***C′′***, Higher-magnification views of boxed areas in ***C***. Arrows point to GFP^+^ Mcm2^+^Ki67^+^ cells, and arrowheads point to GFP^+^ Mcm2^+^Ki67^–^ cells. ***D***, Quantification (mean ± SEM) of electroporated cells expressing Mcm2 and/or Ki67 at E14.0. *p < 0.01; **p < 0.0002. ***E***, Schematics of electroporation strategy combined with the injection of nucleoside analogs. Pregnant dams received a BrdU pulse 1.5 h before dissection, and an EdU pulse 0.5 h before dissection. ***F***, ***G***, Costaining of BrdU (blue) and EdU (red) at E14.0 after a dual-pulse labeling strategy on PCIG (***F***) and PCIG-FL Sox9 (***G***) electroporated brains (green). ***F′***, ***F′′***, Higher-magnification views of boxed areas in ***F***. ***G′***, ***G′′***, Higher-magnification views of boxed areas in ***G***. Arrows point to GFP^+^BrdU^+^EdU^–^ cells, arrowheads point to GFP^+^BrdU^+^EdU^+^ cells, and asterisks point to GFP^+^BrdU^–^EdU^–^ cells. ***H***, Quantification (mean ± SEM) of Ts and Tc duration based on dual-pulse labeling strategy. For Tc calculation, the GF of labeled cells was measured using Mcm2 and Ki67 markers. *p < 0.01; **p < 0.0002. Scale bars: ***B***, ***C***, ***F***, ***G***, low magnification, 20 μm; ***B***, ***C***, ***F***, ***G***, boxed areas, 10 μm.

Although Ki67 is considered a broad cell cycle marker, its expression is largely absent in early G1 phase and in quiescent cells that transitioning from G0/G1 (Miller et al., 2018). Significantly, Sox9 overexpression in a chondrocyte cell line produces accumulations of cells in G0/G1 compared with the control situation (Panda et al., 2001). Therefore, we selected a broader cell cycle marker, the Mcm2, widely expressed in replication-competent cells, including cells in early G1 phase or quiescent stem cells, similar to the adult neural stem cells of the aSVZ (Maslov et al., 2004; Porlan et al., 2014). In the neocortex, the expression of Mcm2 is largely confined to the proliferative regions (Fig. 8*P*), and the proportion of Mcm2^+^/GFP^+^ in the VZ for each condition, Sox9 overexpression, and its control, represents >90% of the total targeted RGCs (PCIG, 97.65 ± 0.63%; PCIG-FL Sox9, 90.25 ± 1.20%; *t*_(6)_ = 5.47, *p* = 0.0016, unpaired *t* test; *n* = 4; Fig. 8*Q*). Double immunostaining with Ki67 and Mcm2 in electroporated embryos at E12.5 analyzed at E14.0 (Fig. 9*A–C*) displayed statistically significant differences in the number of Mcm2^+^Ki67^–^ electroporated cells in the VZ in the Sox9 overexpression condition (PCIG, 4.73 ± 0.79%; PCIG-FL Sox9, 38.30 ± 1.49%; *t*_(6)_ = −19.93, *p* = 1.03 × 10^−6^, unpaired *t* test; *n* = 4; Fig. 9*D*). These data are consistent with the model where Sox9 overexpression in RGCs slows cell cycle progression maintaining part of the cells in early G1 or even keeping those cells in a temporary quiescent state.

To confirm possible alterations in cell cycle length at this age on Sox9 overexpression and estimate the cell cycle duration, we used a dual-pulse thymidine analog labeling strategy according to published methods (Harris et al., 2018). We injected BrdU in E14.0 pregnant females, 1.5 h before the analysis of embryos that were previously electroporated at E12.5 with PCIG or PCIG-FL Sox9 plasmids. A second injection using EdU was administered one-half hour before tissue collection (Fig. 9*E*). GFP^+^ cells present in the VZ were quantified based on BrdU and EdU staining to estimate the duration of S phase (Ts) and Tc (Fig. 9*F*,*G*). As expected, a substantial decrease in the number of double-labeled BrdU/EdU GFP^+^ RGCs and BrdU single-labeled GFP^+^ cells were found on Sox9 overexpression. The Ts calculated as a fraction of double-labeled versus BrdU single-labeled RGCs, as described previously (Martynoga et al., 2005; Harris et al., 2018), was significantly increased in the overexpression condition (PCIG, 5.31 ± 0.39 h; PCIG-FL Sox9, 8.66 ± 0.74 h; *t*_(6)_ = −3.99, *p* = 0.0072, unpaired *t* test; *n* = 4; Fig. 9*H*). The Tc was calculated using the Ts duration and the total number of RGCs in S phase. Because calculation of the Tc assumes that all of the cells are cycling, we can apply a correction factor (growth faction) according to the known proportion of cycling cells. Using Mcm2 expression in GFP^+^ RGCs as a marker of cycling cells, we found that the duration of the cell cycle was three times longer in RGCs overexpressing Sox9 compared with controls (PCIG, 15.14 ± 0.91 h; PCIG-FL Sox9, 49.21 ± 3.91 h; *t*_(6)_ = −8.49, *p* = 0.00,015, unpaired *t* test; *n* = 4; Fig. 9*H*). If we use the percentage of Ki67^+^-targeted RGCs, because Mcm2^+^Ki67^–^ cells could include cells in G0 in addition to early G1, the duration of the cell cycle was almost doubled in time in Sox9-overexpressing RGCs compared with controls (PCIG, 14.41 ± 0.86 h; PCIG-FL Sox9, 28.35 ± 2.25 h; *t*_(6)_ = −5.80, *p* = 0.0011, unpaired *t* test; *n* = 4; Fig. 9*H*). Altogether, these data suggest that elevated levels of Sox9 extend cell cycle duration, thus maintaining progenitors for an extended time in the VZ without undergoing neurogenic divisions.

### Sox9-overexpressing RGCs engage in neurogenic divisions at later stages of cortical development

**Figure 10.**
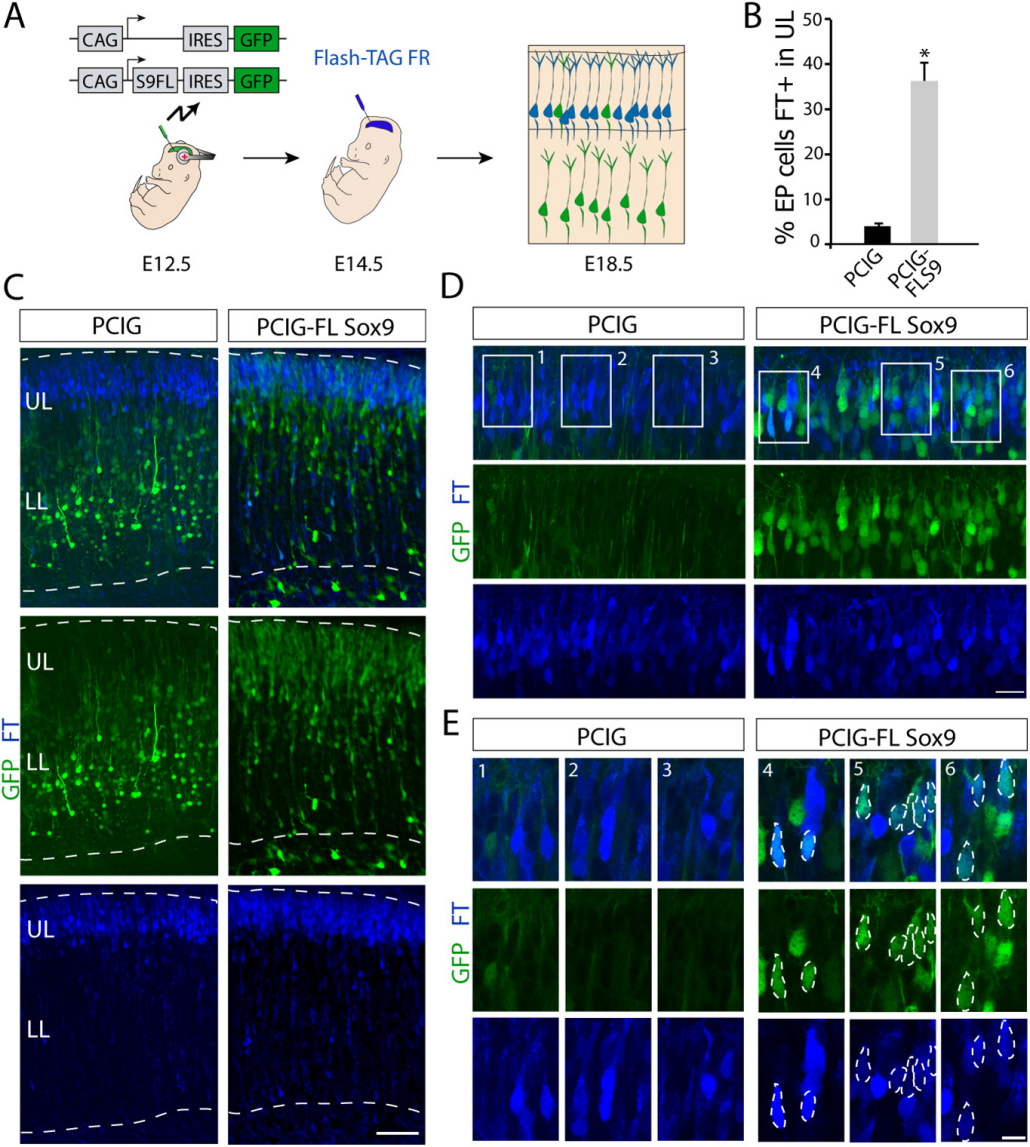
Sox9-overexpressing RGCs engage in neurogenic division at later ages. ***A***, Schematics of the experimental strategy combining electroporation and FlashTag technologies. E12.5 embryos were electroporated with the PCIG or PCIG-FL Sox9 plasmids (green). Forty-eight hours after electroporation, embryos were injected with FlashTag (blue). Brains were analyzed at E18.5. ***B***, Quantification (mean ± SEM) of GFP^+^/FT^+^ double-labeled cells in the CP at E18.5 in both conditions. *p < 0.002. ***C***, Confocal image of E18.5 brains after electroporation and FT injection at described time points. ***D***, Higher-magnification view of ***C*** showing the FT-labeled cells area. ***E***, Higher-magnification view of boxed areas in ***D***. Dotted outline points to examples of GFP^+^/FT^+^ double-labeled cells. Scale bars: ***C***, 100 µm; ***D***, 20 µm; ***E***, 10 µm.

Our data suggest that Sox9 expression levels affect RGC proliferation and their neurogenic potential, such that RGCs expressing high levels of Sox9 during early stages of neocortical neurogenesis have an extended cell cycle duration compared with RGCs with lower Sox9 levels. This would predict that these progenitors expressing high levels of Sox9 would generate neurons mainly at later stages of corticogenesis. To test this hypothesis, we designed an experiment to target RGCs *in vivo* at two different embryonic stages. We first conducted *in utero* electroporation at E12.5 to coexpress Sox9 and GFP or only GFP in RGCs. Forty-eight hours after the first surgery, we performed an intraventricular injection of a carboxyfluorescein ester also known as FlashTag (Telley et al., 2016; Govindan et al., 2018; Fig. 10*A*). FlashTag injection labels RGCs lining the lateral ventricle at E14.5, including those RGCs targeted at E12.5 by *in utero* electroporation that are still in contact with the ventricle. Because Sox9 overexpression at E12.5 causes the accumulation of many RGCs in close contact with the ventricle, visualized 48 h later, those cells would be potentially labeled by FlashTag at the time of the injection. Cells labeled with both GFP and FlashTag are progenitors that were in contact with the ventricle at both E12.5 and E14.5, whereas GFP^+^ cells that are not labeled with FlashTag will include cells that left the VZ by E14.5 like postmitotic neurons. We then analyzed the cell distribution of the offspring at E18.5. In controls, most GFP^+^ cells were FlashTag^–^ and accumulated in the lower part of the CP, while FlashTag^+^/GFP^–^ cells (blue) were largely located in the upper CP (Fig. 10*B*,*C*). Following Sox9 overexpression, a substantial number of GFP^+^ cells occupied the upper CP, the same area also occupied by FlashTag^+^ cells (Fig. 10*C*,*D*). Analysis of single optical sections confirmed colabeling of GFP and FlashTag in these cells (PCIG FT^+^ in UL, 4.09 ± 0.47%; PCIG-FL Sox9 FT^+^ in UL, 36.36 ± 4.17%; *t*_(4)_ = −7.69, *p* = 0.0015, unpaired *t* test; *n* = 3; Fig. 10*B*,*D*,*E*), substantiating that those neurons were generated from RGCs targeted at E12.5 and still present in the VZ at E14.5. We thus conclude that RGCs expressing high levels of Sox9 are maintained longer at the VZ before undergoing neurogenic divisions.

### Sox9-overexpressing RGCs produce neurons destined for upper cortical layers

**Figure 11.**
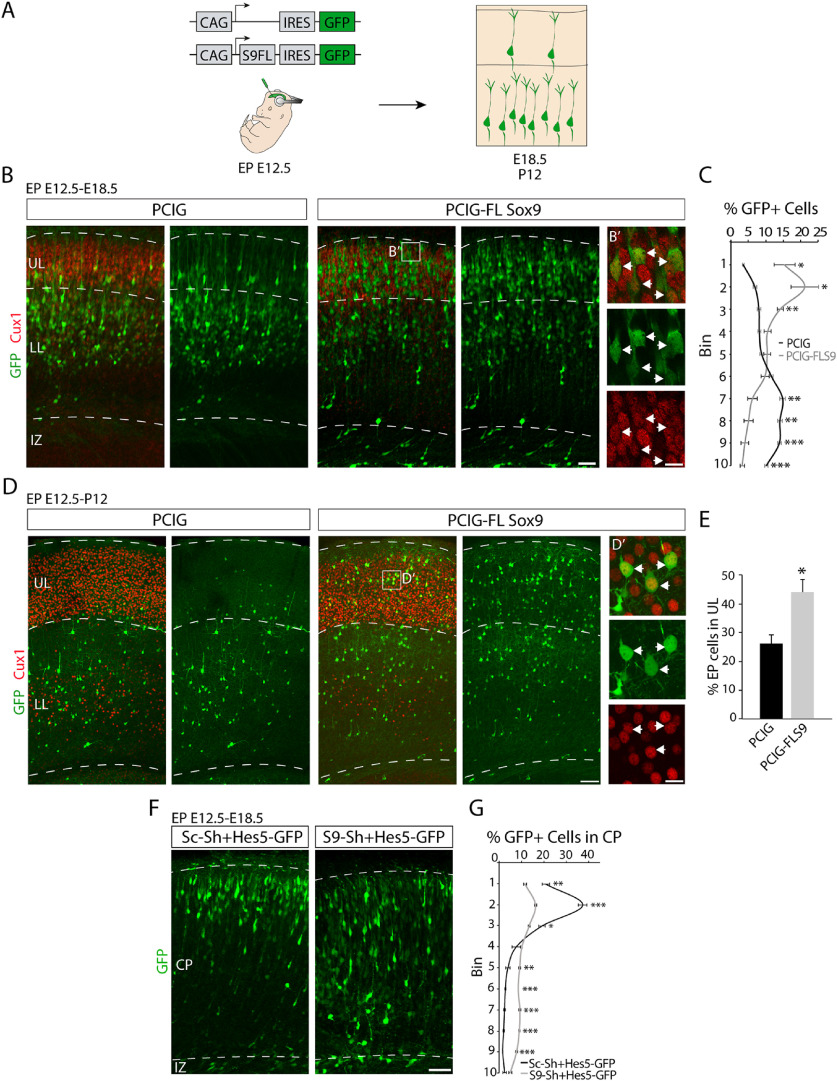
Increased upper layer production on Sox9 overexpression at early ages. ***A***, Illustration of electroporation strategy. E12.5 embryos were electroporated with PCIG or PCIG-FL Sox9 constructs (green). Brains were analyzed at E18.5 or P12. ***B***, Immunostaining for Cux1 (red) on E18.5 brains after E12.5 electroporation of both conditions (green). ***B′***, Higher-magnification view of the boxed area in ***B***. Arrows point to examples of PCIG-FL Sox9 electroporated cells expressing Cux1 at E18.5. ***C***, Quantification (mean ± SEM) of the distribution of electroporated cells within the CP at E18.5. For quantification, the CP was divided into 10 equal-sized bins (enumerated 1–10 from basal to apical). *p < 0.02, **p < 0.005; ***p < 0.0005. ***D***, Immunostaining for Cux1 (red) on P12 brains after E12.5 electroporation of both conditions (green). ***D′***, Higher-magnification view of the boxed area in ***D***. Arrows point to examples of PCIG-FL Sox9 electroporated cells expressing Cux1 at P12. ***E***, Quantification (mean ± SEM) of the distribution of electroporated cells within the CP at P12. For quantification, the CP was divided into two areas, the bottom and top layers of the neocortex. *p < 0.02. ***F***, Confocal image of E18.5 brains after electroporation at E12.5 with sc-shRNA^+^Hes5^–^GFP (control) and Sox9^–^shRNA^+^Hes5^–^GFP plasmid (knockdown). ***G***, Quantification (mean ± SEM) of the distribution of electroporated cells within the CP at E18.5. For quantification, the CP was divided into 10 equal-sized bins (enumerated 1–10 from basal to apical). *p < 0.02; **p < 0.005; ***p < 0.0005. Scale bars: ***B***, ***D***, ***G***, 100 µm; ***B′***, ***D′***, 20 μm.

To further investigate the fate of neurons derived from RGCs with high Sox9 expression levels, we quantified their laminar positions. We conducted *in utero* electroporation at E12.5 to overexpress Sox9 with GFP or GFP alone and determined the laminar position of the neuronal offspring of electroporated RGCs at E18.5 and P12 (Fig. 11*A*). Following Sox9 overexpression, more neurons occupied the upper part of the CP compared with controls at E18.5, expressing the upper layer marker Cux1 (PCIG vs PCIG-FL Sox9: bin 1, *p* = 0.0079; bin 2, *p* = 0.0111; bin 3, *p* = 0.0008; bin 7, *p* = 0.0018; bin 8, *p* = 0.0008; bin 9, *p* = 0.0002; bin 10, *p* = 0.0001; unpaired *t* test; *n* = 4; Fig. 11*B*,*C*). At P12, when cell migration is complete, these neurons expressing Cux1 occupied upper neocortical cell layers (PCIG, 26.14 ± 3.14%; PCIG-FL Sox9, 44.1 ± 4.48%; *t*_(6)_ = 3.28, *p* = 0.0167, unpaired *t* test; *n* = 4; Fig. 11*D*,*E*).

Since Sox9 overexpression increased the production of neurons occupying upper cortical layers, we also analyzed the influence of Sox9 downregulation on the laminar localization of Hes5^+^ RGC offspring. To this end, we performed E12.5 *in utero* electroporation with the same Sox9 or scrambled shRNA used above (Fig. 7*I–L*) together with a Hes5-GFP vector. The analysis of the laminar position of targeted cells at E18.5 showed important changes in the distribution of the produced neurons. A significant reduction in the number of GFP^+^ neurons was found in the upper part of the CP compared with the control (sc-shRNA vs S9-shRNA: bin 1, *p* = 0.002; bin 2, *p* = 3.83 × 10^−5^; bin 4, *p* = 0.009; bin 5, *p* = 0.0022; bin 6, *p* = 2.61 × 10^−6^; bin 7, *p* = 2.82 × 10^−5^; bin 8, *p* = 2.28 × 10^−6^; bin 9, *p* = 28.82 × 10^−6^; unpaired *t* test, *n* = 4; Fig. 11*F*,*G*). These results are consistent with the model that the cortical VZ contains a subset of RGCs expressing high levels of Sox9, which are maintained as progenitors during early stages of neurogenesis and generate upper layer neurons later in development.

## Discussion

We provide here evidence that RGCs at early stages of cortical development are molecularly diverse and show differences in their cell cycle behavior. Specifically, we identified two types of progenitors that differ in their cell cycle behavior and the expression of 11 genes. We demonstrate that one of these genes, Sox9, is expressed at different levels in RGCs and that RGCs with high expression levels of Sox9 are preferentially maintained as RGCs during a time point when RGCs with lower Sox9 levels undergo neurogenic divisions. Our functional experiments provide strong evidence that Sox9 levels play a critical role in regulating the decision of neocortical progenitors to be maintained as RGCs or to undergo neurogenic divisions. Our data suggest that high levels of Sox9 extend the cell cycle duration of RGCs, delaying neurogenesis during early stages of corticogenesis, to then generate neurons during later stages of neocortical development.

Roles for Sox9 in the control of neural stem cells maintenance and differentiation have already been described in several parts of the brain, including the cerebellum (Vong et al., 2015), spinal cord (Stolt et al., 2003), and the postnatal subventricular zone (Cheng et al., 2009). Our data indicate that Sox9 is important in the regulation of neural stem cell behavior in the early embryonic neocortex. Interestingly, Sox9 is expressed widely in all RGCs, and lineage-tracing experiments using Sox9-CreERT2 mice showed that neurons of all neocortical cell layers were labeled (Kaplan et al., 2017). These data suggest that the expression levels of Sox9 in RGCs are likely more important than presence or absence. Indeed, a similar dose-dependent regulation of RGC neurogenic behavior has been described for Sox2, another transcription factor of the same family as Sox9 (Hagey and Muhr, 2014). Higher levels of Sox2 have been found in slow-cycling RGCs, and Sox2 overexpression by *in utero* electroporation reduces their proliferation rate.

In the adult subventricular zone, increased Sox9 levels are a marker of quiescent NSCs (Llorens-Bobadilla et al., 2015; Belenguer et al., 2021). However, these progenitors need to be activated to generate neurons at appropriate times, similar to the activation of VZ progenitors to switch from a self-renewal state during early phases of corticogenesis to neurogenic divisions during late stages of cortical development. It is noteworthy that one of the parameters described as changing in the transition between self-renewing to neurogenic progenitors is the duration of S phase (Arai et al., 2011; Taverna et al., 2014). Our functional analysis confirmed that RGCs overexpressing Sox9 present enlarged S phase duration, compared with the control situation, and progress more slowly through the cell cycle. However, eventually these cells engage in neurogenic divisions producing an increased number of UL neurons that are able to properly migrate, suggesting that elevated Sox9 levels are not affecting the migration process. How the neurogenic brake is overcome, and which molecular pathways are involved is unclear. One interesting molecular pathway is the Yap/Hippo pathway. Hippo signaling has been related to tissue homeostasis, organ size, and stem cell self-renewal (Pan, 2010; Schlegelmilch et al., 2011; Zanconato et al., 2016). A recent report has described that Yap1/Taz promotes NSC maintenance in the cortex and the production of corticocortical neurons at the expense of corticofugal neurons (Mukhtar et al., 2020). Interestingly, Sox9 has been described as a downstream target of Yap1 signaling and Yap1 targeting microRNAs, induced by Sox9, post-transcriptionally repressing Yap1 expression, contributing to a negative feedback loop (Wang et al., 2019). Yap1 expression is high at early corticogenesis stages, and its expression decreases at mid-corticogenesis (E14 to E16.5) to increase again at early postnatal ages (Mukhtar et al., 2020). It will be interesting to test in the future whether the decrease in Yap1 expression during mid-neurogenesis is critical for determining Sox9 levels and the neurogenic potential of RGCs.

The extent in which different subpopulations of RGCs contribute directly to the generation of cellular diversity in the neocortex is a controversial topic (Franco et al., 2012; Fuentealba et al., 2015; García-Moreno and Molnár, 2015; Gil-Sanz et al., 2015). The results shown here reveal the existence of molecular heterogeneity in RGCs associated with the production of particular cell types. A previously published scRNA-seq study did not detect such molecular diversity (Telley et al., 2019). However, this study used a different method to label and isolate RGCs; instead of *in utero* electroporation, they used FlashTag technology. Notably, FlashTag has been described as only labeling actively dividing RGCs in M phase, because those cells maintain a large contact surface with the ventricle (Govindan et al., 2018). At early embryonic ages (E12.5), the progeny of these labeled cells rapidly exits the VZ to produce IPs or ENs (Telley et al., 2016, 2019). Thus, 10 h after FlashTag labeling, as conducted in this study, most of the labeled cells were found in the SVZ-IZ. In contrast, our *in utero* electroporation approach at the same ages using different promoter fragments allowed the identification of late neurogenic RGCs that can be visualized in the VZ even 24 h after labeling by electroporation, which produce neurons at later ages. These cells would have been missed in the previous study, given that FlashTag apparently does not label slowly dividing RGCs that would not be in M phase. Notably, our data also show that these progenitors have a specific molecular signature with some genes being more highly expressed, including Sox9. While we describe here a function of Sox9 for the regulation of differential progenitor behavior, the function of the other differentially expressed genes still needs to be investigated.

In summary, we demonstrated here the existence of molecular heterogeneity among neocortical progenitors, which distinguishes RGCs with different neurogenic activity at early ages of cortical development. Among the differentially expressed genes that we found, Sox9 has an instructive role in defining the neurogenic potential of RGCs. The levels of expression of this gene are further associated with the production of distinct cell types, with higher levels of Sox9 instructing the production of upper layer neurons. These results suggest that the delayed neurogenic behavior instructed by Sox9 is important for preventing some RGCs from experiencing early exhaustion, allowing them to generate upper layer neurons at later embryonic ages. Because the differential neurogenic behavior of these RGCs and their particular molecular signature have been analyzed only at one particular age, additional work will be required to fully clarify whether this molecular signature is maintained throughout different stages of cortical development and may even play a role in adult stem cells.
