# Supplementary figures and images for: Differential Expression Levels of Sox9 in Early Neocortical Radial Glial Cells Regulate the Decision between Stem Cell Maintenance and Differentiation
Source: J Neurosci. 2021 Aug 18;41(33):6969–86. doi: 10.1523/JNEUROSCI.2905-20.2021 (PMC8372026; doi:10.1523/JNEUROSCI.2905-20.2021)

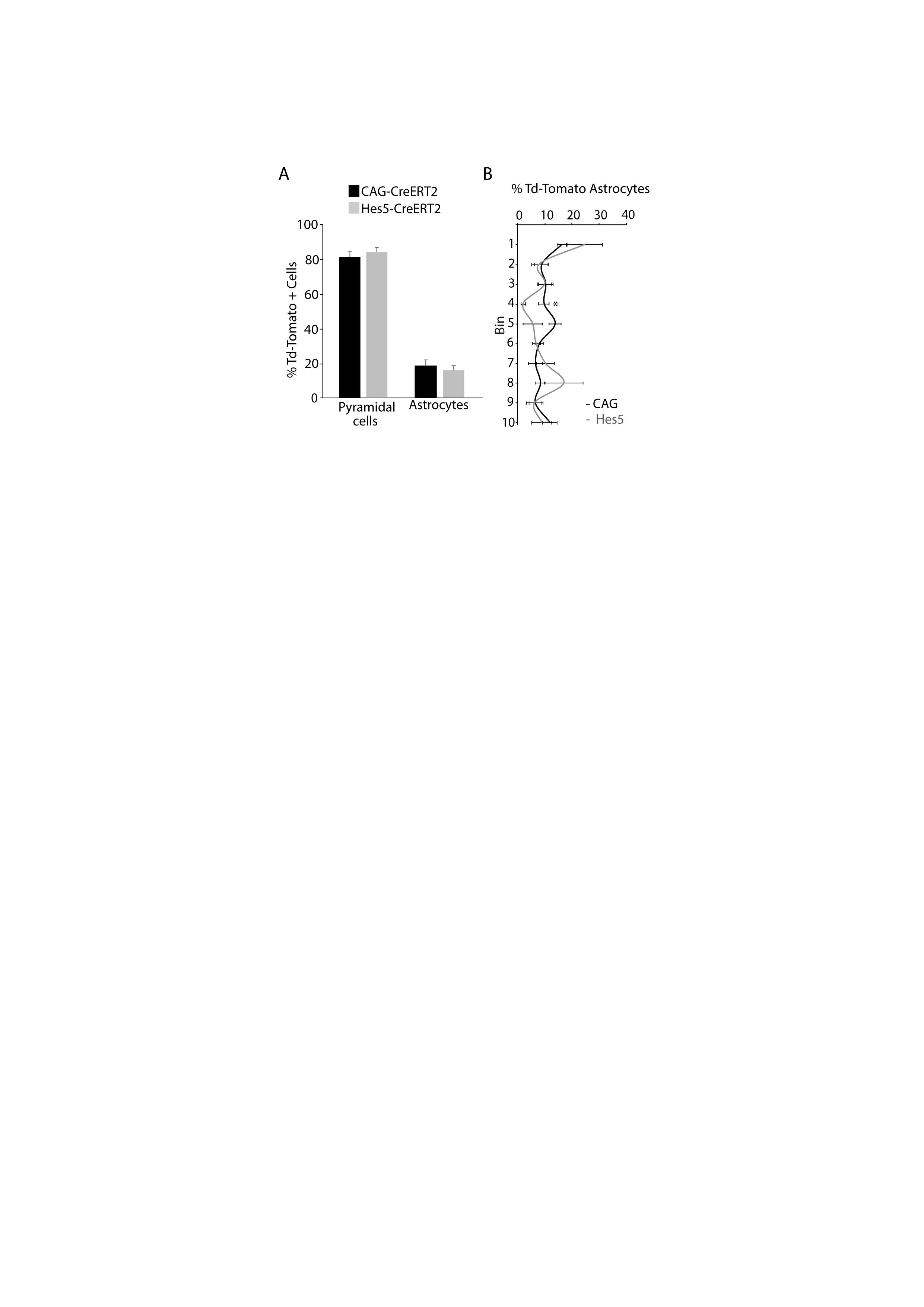

Supplement: Figure 3-1 — Glial versus neural differentiation of RGCs activating Hes5 promoter. A, Quantification (mean ± SEM) of the cell type of td-Tomato+ cells at P20. Ai9 reporter embryos were electroporated with Hes5-CreERT2 or CAG-CreERT2 plasmids. Morphological features were used to distinguish pyramidal cells and astrocytes. B, Quantification (mean ± SEM) of the distribution of td-Tomato+ astrocytes within the CP at P20. Ai9 reporter embryos were electroporated with Hes5-CreERT2 or CAG-CreERT2 plasmids. For quantification, the CP was divided into 10 equal-sized bins (enumerated 1–10 from basal to apical). *p < 0.05. Download Figure 3-1, TIF file. [file ns-JN-RM-2905-20-s01.tif]
